# A Class of Environmental and Endogenous Toxins Induces *BRCA2* Haploinsufficiency and Genome Instability

**DOI:** 10.1016/j.cell.2017.05.010

**Published:** 2017-06-01

**Authors:** Shawn Lu Wen Tan, Saakshi Chadha, Yansheng Liu, Evelina Gabasova, David Perera, Karim Ahmed, Stephanie Constantinou, Xavier Renaudin, MiYoung Lee, Ruedi Aebersold, Ashok R. Venkitaraman

**Affiliations:** 1 Medical Research Council Cancer Unit, University of Cambridge, Hills Road, Cambridge CB2 0XZ, UK; 2 Department of Biology, Institute of Molecular Systems Biology, Eidgenössische Technische Hochschule, 8093 Zürich, Switzerland; 3 Faculty of Science, University of Zürich, 8006 Zürich, Switzerland

**Keywords:** BRCA2, formaldehyde, acetaldehyde, aldehyde, replication stress, R-loop, induced haploinsufficiency, proteasomal degradation, SWATH-MS

## Abstract

Mutations truncating a single copy of the tumor suppressor, *BRCA2*, cause cancer susceptibility. In cells bearing such heterozygous mutations, we find that a cellular metabolite and ubiquitous environmental toxin, formaldehyde, stalls and destabilizes DNA replication forks, engendering structural chromosomal aberrations. Formaldehyde selectively depletes BRCA2 via proteasomal degradation, a mechanism of toxicity that affects very few additional cellular proteins. Heterozygous *BRCA2* truncations, by lowering pre-existing BRCA2 expression, sensitize to *BRCA2* haploinsufficiency induced by transient exposure to natural concentrations of formaldehyde. Acetaldehyde, an alcohol catabolite detoxified by *ALDH2*, precipitates similar effects. Ribonuclease H1 ameliorates replication fork instability and chromosomal aberrations provoked by aldehyde-induced *BRCA2* haploinsufficiency, suggesting that BRCA2 inactivation triggers spontaneous mutagenesis during DNA replication via aberrant RNA-DNA hybrids (R-loops). These findings suggest a model wherein carcinogenesis in *BRCA2* mutation carriers can be incited by compounds found pervasively in the environment and generated endogenously in certain tissues with implications for public health.

## Highlights

• Pervasive natural aldehydes selectively deplete BRCA2 and a few additional proteins
• Inherited *BRCA2* mutations sensitize to aldehyde-induced *BRCA2* haploinsufficiency
• BRCA2 haploinsufficiency causes genomic instability via unscheduled R-loops
• Cellular or environmental aldehydes may incite cancer in *BRCA2* mutation carriers

## Graphical Abstract

**Figure:**
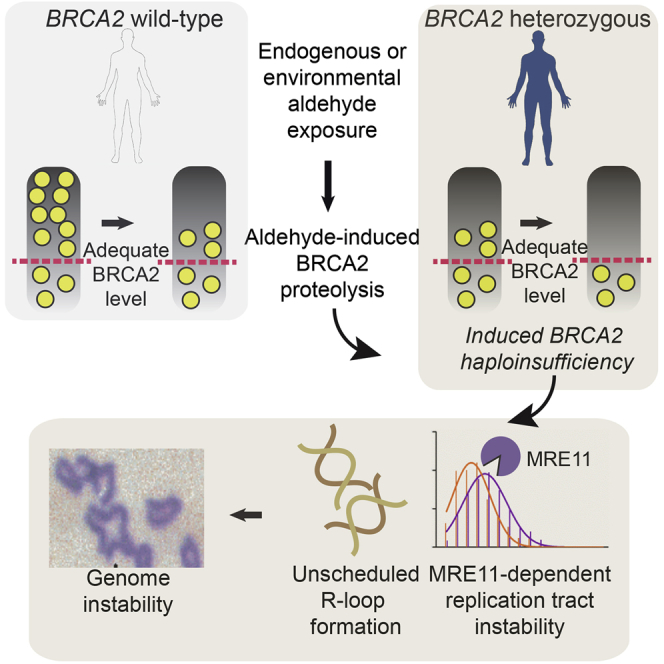

## Teaser

Environmental and endogenous aldehydes contribute to genome instability and potentially tumorigenesis through selective degradation of BRCA2.

## Introduction

Inherited germline mutations affecting a single copy of the *BRCA2* tumor suppressor gene predispose to cancers of the breast, ovaries, pancreas, prostate, and other organs (Breast Cancer Linkage Consortium, 1999). Human *BRCA2* encodes a nuclear-localized protein of 3,418 residues, which is essential for the maintenance of chromosome integrity, through functions in homology-directed DNA repair, in stabilizing stalled DNA replication forks, or in mitotic cell division (reviewed in Venkitaraman, 2014). Aberrations in chromosome structure and increased sensitivity to genotoxic agents typically occur after bi-allelic *BRCA2* disruption in murine or human cells, rather than with mutations affecting a single allele (Connor et al., 1997, Patel et al., 1998, Skoulidis et al., 2010). Organ development and function is grossly normal in genetically engineered mice heterozygous for mutant *BRCA2* alleles (Connor et al., 1997, Friedman et al., 1998, Ludwig et al., 1997, Sharan et al., 1997, Suzuki et al., 1997), as is homology-directed DNA repair in multiple tissues (Kass et al., 2016). What promotes carcinogenesis in carriers of heterozygous *BRCA2* mutations is therefore unclear.

Inherited missense mutations in *BRCA2* may act dominantly to suppress the wild-type allele (Jeyasekharan et al., 2013). However, the most prevalent *BRCA2* alleles that confer a clinically significant risk of cancer susceptibility encode nonsense or frameshift mutations, which prematurely truncate the BRCA2 protein (Rebbeck et al., 2015) (Breast Cancer Information Core [BIC] database, https://research.nhgri.nih.gov/bic/). These truncating mutations include the *6174delT* mutation prevalent among the Ashkenazim (Neuhausen et al., 1996), the pathogenic truncation *3036del4* (BIC database) representative of variants associated with breast and ovarian cancer, or carboxyl (C)-terminal truncating mutations like *7691insAT* or *9900insA* implicated in Fanconi anemia (Howlett et al., 2002). We have investigated the mechanism by which heterozygosity for such *BRCA2* truncating mutations may promote carcinogenesis.

Here, we report that exposure to naturally occurring concentrations of formaldehyde or acetaldehyde selectively unmasks genomic instability in cells heterozygous for multiple, clinically relevant, truncating *BRCA2* mutations. These agents are not only widespread in our environment, but also accumulate endogenously in certain tissues via critical metabolic reactions such as oxidative demethylation or alcohol catabolism (Harris et al., 2003, Roy and Bhagwat, 2007, Shi et al., 2004). Aldehydes selectively deplete BRCA2 via proteasomal degradation, rendering *BRCA2* heterozygous cells vulnerable to induced *BRCA2* haploinsufficiency. Induced *BRCA2* haploinsufficiency provokes chromosomal aberrations through DNA replication fork stalling and the MRE11-dependent degradation of nascent DNA, via the unscheduled formation of RNA-DNA hybrids. These previously unrecognized cellular effects of aldehydes may potentiate genome instability and promote tissue-specific cancer evolution in patients who inherit pathogenic *BRCA2* truncations, with implications for cancer biology and public health.

## Results

### Formaldehyde Stalls DNA Replication and Triggers Strand Breakage

**Figure 1:**
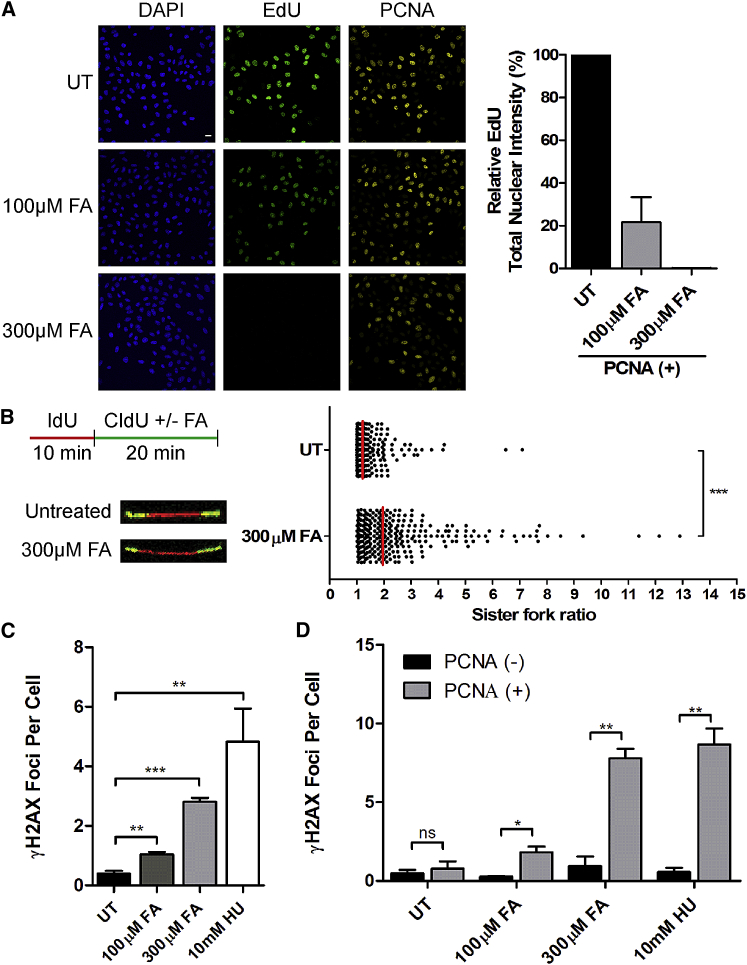
Formaldehyde Stalls DNA Replication and Induces Strand Breakage in Dividing Cells (A) Immunofluorescence images of HeLa Kyoto cells labeled with EdU (1 hr) after 2 hr formaldehyde (FA) treatment. UT, untreated. Scale bars, 20 μm. The histogram quantifies the mean ± SEM of total EdU nuclear intensities, n = 3. (B) DNA fiber analysis comparing sister fork symmetry. The experimental setup and representative images are shown. The scatterplot compares the ratio of sister-fork tract lengths (see the STAR Methods) between untreated (UT) and FA-treated conditions. Red lines represent the median, n = 3. Statistical significance was determined by the Mann-Whitney t test, n = 3. (C) Mean ± SEM of γH2AX foci per cell 3 hr after indicated treatments. Greater than or equal to 1,500 cells were analyzed per condition. Statistical significance was determined by the two-tailed Student’s t test, n = 4. (D) Mean ± SEM of γH2AX foci per cell in PCNA^+^ versus PCNA^−^ cells after 3-hr exposure to FA or HU. Statistical significance was determined by the two-tailed Student’s t test, n = 3.

Formaldehyde, a widespread environmental toxin, occurs at 50–100 μM in human blood (Heck et al., 1985, Luo et al., 2001) and reacts readily with both proteins and DNA to generate adducts and cross-linkages (Huang et al., 1992, Lu et al., 2010, Solomon and Varshavsky, 1985) expected to impede DNA transactions in the cell nucleus. Mice doubly deficient in the Fanconi anemia protein FANCD2 and in the formaldehyde-catabolizing enzyme ADH5 sustain DNA damage and retarded growth (Pontel et al., 2015). To characterize the effect of formaldehyde on DNA replication, HeLa Kyoto cells exposed to formaldehyde for 2 hr were labeled with 5-ethynyl 2′-deoxyuridine (EdU) to measure DNA synthesis and co-stained for the S-phase marker, proliferating cell nuclear antigen (PCNA). PCNA-positive cells exhibit a dose-dependent decrease in EdU incorporation when exposed to 100 μM or 300 μM formaldehyde (Figure 1A). DNA fiber analysis after pulse labeling with 5-iodo-2′-deoxyuridine (IdU) and then 5-chloro-2′-deoxyuridine (CldU) shows that formaldehyde significantly increases the asymmetry of sister replication fork tracts emanating from the same origin of replication (Figure 1B), a consistent marker of replication fork stalling (Schwab et al., 2015), from a median ratio of 1.18 in untreated (UT) cells to 1.87 following formaldehyde (FA) treatment (p < 0.001, Mann-Whitney t test). Formaldehyde also increases staining for γH2AX (Figure 1C), a marker of DNA breakage. Notably, γH2AX foci accumulate prominently in PCNA-positive cells (Figure 1D), suggesting that formaldehyde selectively causes DNA damage during DNA replication. The DNA synthesis inhibitor, hydroxyurea (HU), elicits similar effects (Figures 1C and 1D). Thus, formaldehyde stalls DNA replication and triggers strand breakage in dividing cells.

### Heterozygous BRCA2 Truncations Sensitize Selectively to Formaldehyde-Induced Replication Stress

**Figure 2:**
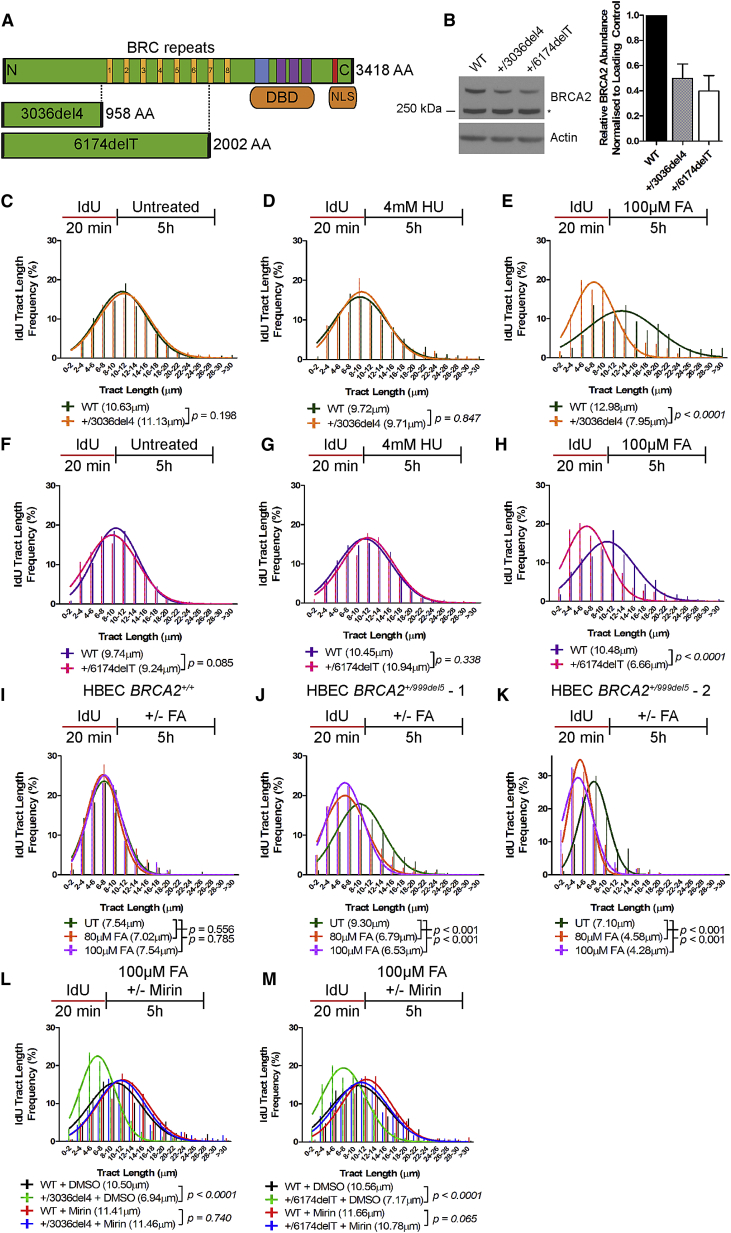
Heterozygosity for *BRCA2* Truncating Mutations Selectively Sensitizes Cells to Formaldehyde-Induced Replication Stress (A) The pathogenic *BRCA2* truncating mutants used in this work. DBD, DNA binding domain; NLS, nuclear localization signal. (B) BRCA2 protein levels in HeLa Kyoto cells. ^∗^Denotes non-specific bands. The histogram plots normalized BRCA2 band intensities (mean ± SEM, n = 3). (C–E) IdU tract length frequency distributions of wild-type HeLa Kyoto versus *BRCA2*^+/3036del4^ heterozygous cells (C) without treatment, (D) with 4 mM HU, and (E) with 100 μM FA. (F–H) IdU tract length frequency distributions of wild-type HeLa Kyoto versus *BRCA2*^+/6174delT^ heterozygous cells (F) without treatment, (G) with 4 mM HU, and (H) with 100 μM FA. (I) IdU tract length frequency distributions of *BRCA2*^*+/+*^ HBECs treated with or without FA for 5 hr. (J) IdU tract length frequency distributions of *BRCA2*^*+/999del5*^ - 1 HBECs treated with or without FA for 5 hr. (K) IdU tract length frequency distributions of *BRCA2*^*+/999del5*^ - 2 HBECs treated with or without FA for 5 hr. (L and M) IdU tract length frequency distributions of HeLa Kyoto cells after treatment with FA in the presence (100 μM) or absence (DMSO) of Mirin. Results in (C)–(M) represent at least two independent experiments. See also Figures S1 and S2.

From the HeLa Kyoto cell line (that is diploid for *BRCA2*) (Adey et al., 2013), we created cells heterozygous for the clinically relevant *BRCA2* truncating mutations *6174delT* and *3036del4* using CRISPR/Cas9-mediated genome engineering (Ran et al., 2013). While *6174delT* encodes a frameshift mutation leading to a premature stop codon at amino acid 2002, the *3036del4* frameshift mutation similarly truncates the BRCA2 protein at amino acid 958 (Figure 2A). Both mutations cause cancer predisposition in humans (Ramus and Gayther, 2009).

**Figure S1:**
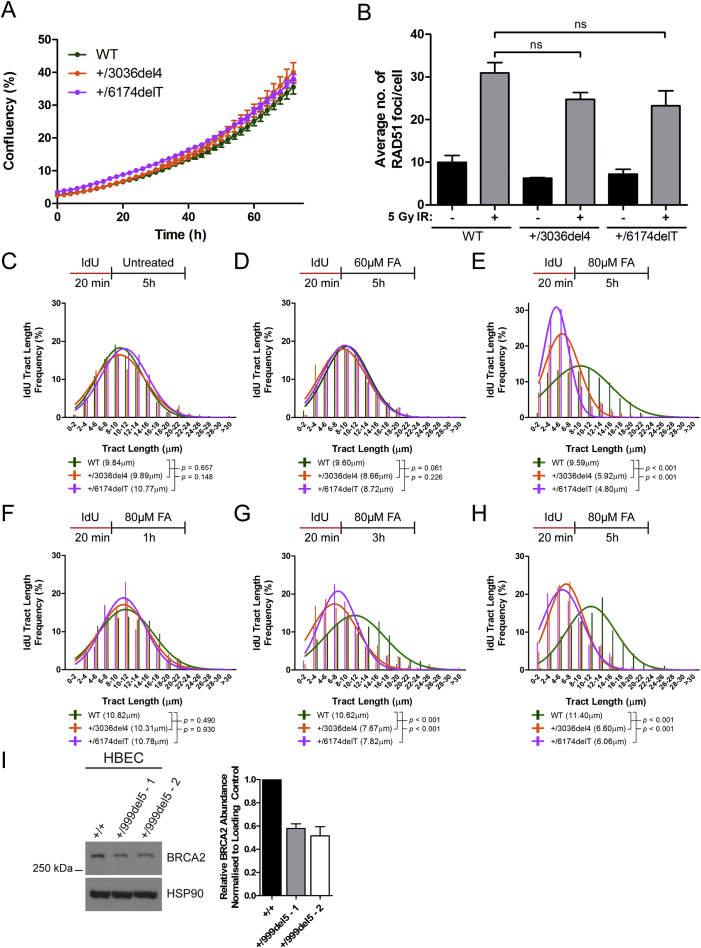
Related to Figure 2 (A) Growth curves of HeLa Kyoto cells. Mean ± SD from nine fields of view. (B) Number of RAD51 foci per cell in HeLa Kyoto cells 3h after exposure to 5 Gy ionising radiation. Mean ± SEM, n = 3. (C–E) IdU tract length frequency distributions in HeLa Kyoto cells after treatment with different doses of FA for 5h. (F–H) IdU tract length frequency distributions in HeLa Kyoto cells after treatment with 80μM FA for different lengths of time. (I) BRCA2 protein levels in *BRCA2*^+/+^ or *BRCA2*^+/999del5^ human breast epithelial cells. Normalized BRCA2 band intensities are represented in the histogram. Mean ± SEM, n = 2.

Heterozygosity for the *6174delT* or *3036del4* mutations lowered wild-type BRCA2 protein expression to 40%–60% of that in parental cells, although the truncated proteins were undetectable (Figure 2B). The proliferation of the heterozygous cell lines was indistinguishable from the parental cells (Figure S1A), and there was no significant change (Figure S1B) in the formation of RAD51 foci at sites of DNA damage after 5 Gy ionizing radiation (IR), a key surrogate marker for BRCA2’s functions in homologous DNA recombination (Yuan et al., 1999) and replication fork stabilization (Petermann et al., 2010, Schlacher et al., 2011). Our findings are in accord with previous reports in which *BRCA2* heterozygosity impairs neither cell proliferation nor the control of RAD51.

Bi-allelic *BRCA2* inactivation causes cellular sensitivity to replication stress provoked by HU, accompanied by the destabilization of DNA replication forks (Lomonosov et al., 2003, Schlacher et al., 2011). By contrast, haploinsufficiency for the related cancer suppressor, *BRCA1*, suffices to cause defects in stalled replication fork repair or integrity following HU exposure (Pathania et al., 2014). We therefore compared the effects of HU with formaldehyde in *BRCA2* heterozygous cells using the DNA fiber assay to measure IdU-labeled tract length.

Without treatment, the median length of IdU-labeled replication tracts in *BRCA2 6174delT* or *3036del4* heterozygous cells was similar to that in parental cells (Figures 2C and 2F), confirming *BRCA2* heterozygosity does not affect processive DNA replication. There was also no significant change after HU exposure (Figures 2D and 2G). By contrast, formaldehyde treatment at 100 μM significantly shortened IdU-labeled replication tracts in cells heterozygous for *BRCA2 6174delT* or *3036del4* compared to the parental cells (Figures 2E and 2H).

Exposure to as little as 80 μM formaldehyde (Figures S1C–S1E) for no more than 3 hr (Figures S1F and S1G) provokes replication tract shortening in *BRCA2 6174delT* or *3036del4* heterozygous cells, but not parental controls. Similar anomalies occur in immortalized human breast epithelial cells (HBECs) from patients heterozygous for the pathogenic *BRCA2*^999del5^ truncation (Rubner Fridriksdottir et al., 2005), in contrast to wild-type controls (Figures 2I–2K). Thus, transient exposure to naturally occurring formaldehyde concentrations selectively provokes DNA replication fork instability in *BRCA2* heterozygous cells derived from a target tissue for carcinogenesis in mutation carriers but not in wild-type controls, speaking to the physiological relevance of our findings.

**Figure S2:**
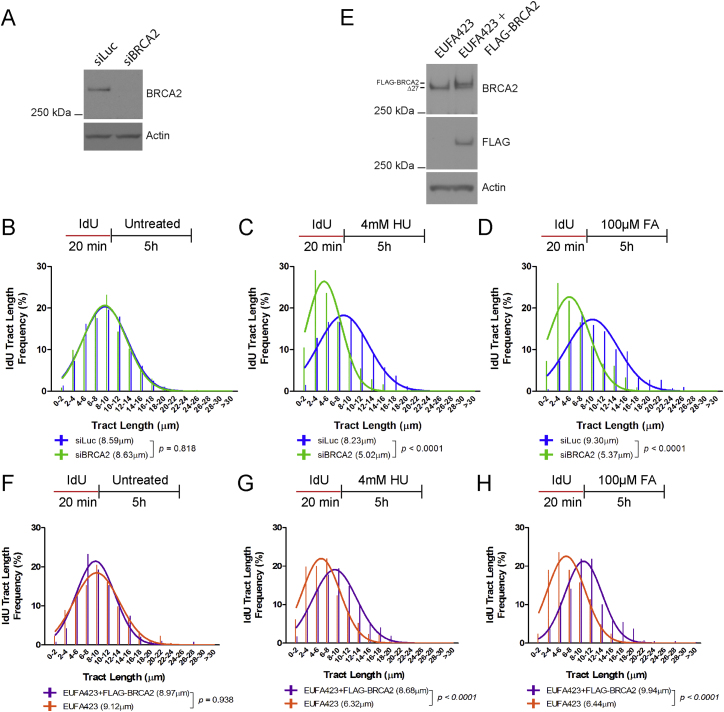
Related to Figure 2 (A) BRCA2 protein levels in wild-type HeLa Kyoto cells 24h after transfection with 50nM short interfering RNAs to Luc (siLuc) or BRCA2 (siBRCA2). (B–D) IdU tract length frequency distributions of wild-type HeLa Kyoto cells treated with (siBRCA2) or without (siLuc) BRCA2 knockdown under the indicated conditions. (E) BRCA2 protein levels in EUFA423 cells and EUFA423 cells complemented with FLAG-BRCA2. Δ27 refers to the exon 27 truncated variant from the *BRCA2 9900insA* mutant allele. The truncated product from the *BRCA2 7691insAT* allele was not detectable. (F–H) IdU tract length frequency distributions of EUFA423 cells, with or without FLAG-BRCA2 complementation, treated under the indicated conditions. Results in (B)–(D) and (F)–(H) represent two independent experiments.

In contrast to *BRCA2* heterozygosity, depletion of BRCA2 from HeLa Kyoto parental cells with short interfering RNA (siRNA) (Figure S2A) destabilizes DNA replication forks stalled by exposure either to HU or to formaldehyde (Figures S2B–S2D). Similar experiments using the BRCA2-deficient cell line, EUFA423 (that harbors inactivating bi-allelic mutations that truncate one *BRCA2* allele at exon 15 [*7691insAT*] and the second, at exon 27 [*9900insA*]) (Howlett et al., 2002), or control EUFA423 cells stably reconstituted (Figure S2E) with full-length FLAG epitope-tagged BRCA2 (Hattori et al., 2011, Jeyasekharan et al., 2013), yielded similar results (Figures S2F–S2H). These findings, with prior reports (Lomonosov et al., 2003, Schlacher et al., 2011, Ying et al., 2012), confirm that BRCA2 is dispensable for processive DNA replication but essential to preserve the stability of stalled DNA replication forks, both after exposure to HU as well as formaldehyde.

### Formaldehyde Exposes Stalled DNA Replication Forks in *BRCA2* Heterozygous Cells to MRE11-Dependent Strand Degradation

BRCA2 protects nascent DNA strands at stalled DNA replication forks from degradation by the endonuclease, MRE11 (Schlacher et al., 2011, Ying et al., 2012). Mirin (Dupré et al., 2008), a selective small-molecule inhibitor of MRE11, but not its vehicle, DMSO, significantly inhibits the degradation of IdU-labeled replication tracts after formaldehyde treatment in *BRCA2 3036del4* (Figure 2L) or *6174delT* (Figure 2M) heterozygous cells. This suggests that formaldehyde exposes stalled DNA replication forks in *BRCA2* heterozygous cells to MRE11-dependent strand degradation.

### Selective Proteasomal Degradation of BRCA2 Protein after Formaldehyde Exposure

**Figure 3:**
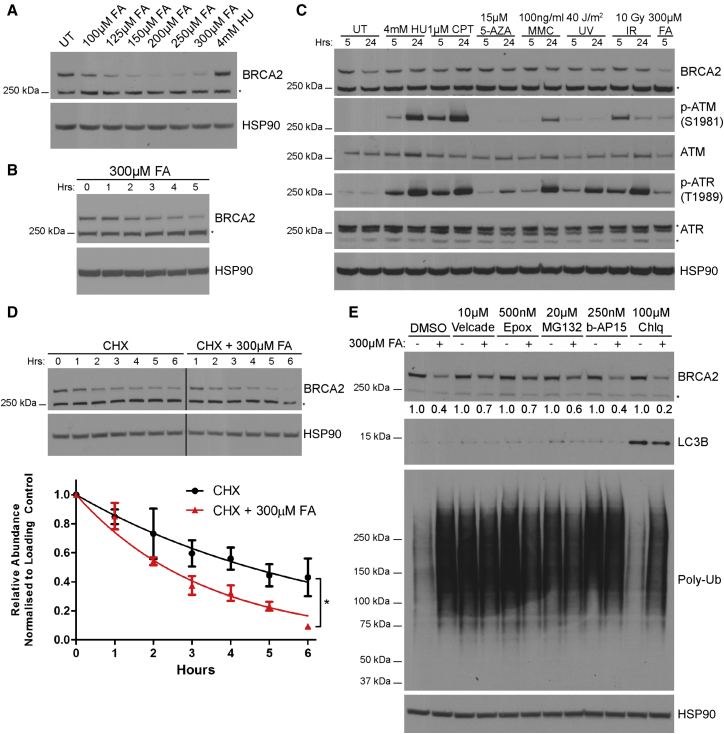
Selective Proteasomal Degradation of BRCA2 Protein after Formaldehyde Exposure (A) BRCA2 protein levels in wild-type HeLa Kyoto cells after 5-hr treatments. (B) BRCA2 protein levels in wild-type HeLa Kyoto cells treated as indicated. (C) BRCA2 protein levels in wild-type HeLa Kyoto cells treated with various DNA damaging agents for the indicated durations. HU, hydroxyurea; CPT, camptothecin; 5-AZA, 5-azacytidine; MMC, mitomycin C; UV, ultraviolet; IR, ionizing radiation; FA, formaldehyde. (D) BRCA2 protein turnover in wild-type HeLa Kyoto cells treated with or without 300 μM FA. Mean ± SEM of BRCA2 band intensities normalized to loading control and 0 hr, n = 3. CHX, cycloheximide. (E) BRCA2 protein levels in wild-type HeLa Kyoto cells pre-treated with various inhibitors for 3 hr prior to addition of 300 μM FA for 3 hr. Normalized BRCA2 band intensities are shown below. Results represent two independent experiments. Epox, epoxomicin; Chlq, chloroquine. See also Figure S3.

**Figure S3:**
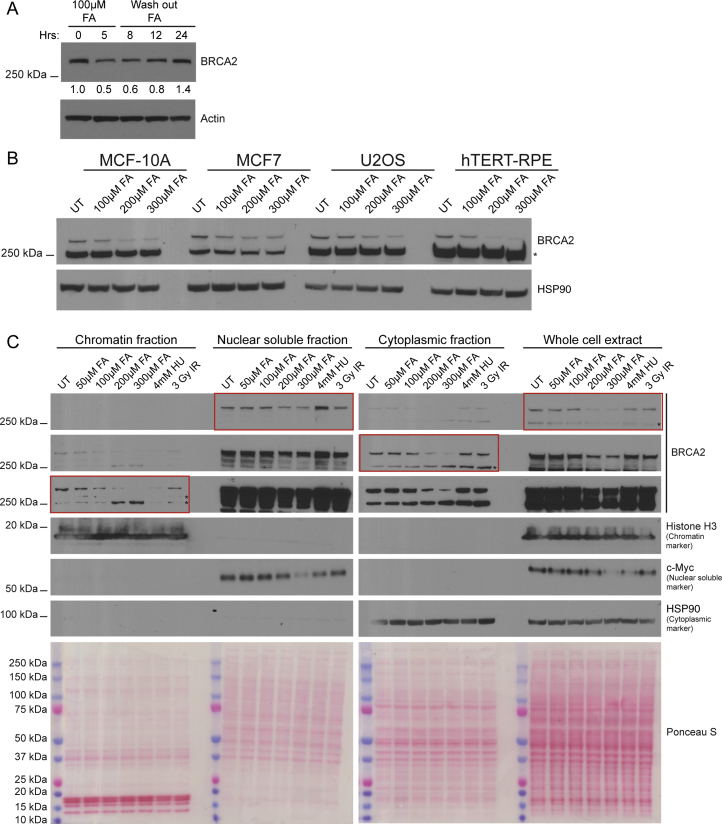
Related to Figure 3 (A) BRCA2 protein levels at the indicated time points in wild-type HeLa Kyoto cells. Cells were treated with 100μM FA for 5h, washed and harvested for western blotting at the indicated time points. Numbers below the BRCA2 blot represent densitometric measurements of BRCA2 band intensities normalized to loading control and the 0h time point. (B) BRCA2 protein levels in cells from four different cell lines after treatment with the increasing doses of formaldehyde for 5h. (C) BRCA2 protein levels in different cellular fractions of wild-type HeLa Kyoto cells after exposure to the indicated treatments for 3h. Different exposures of BRCA2 are shown, with red boxes highlighting the appropriate exposures for the various fractions.

Surprisingly, formaldehyde consistently causes dose-dependent BRCA2 protein depletion over a 5 hr period in cells that are wild-type for *BRCA2*, whereas HU does not (Figures 3A and 3B). This effect is transient (Figure S3A), persisting for 8–12 hr after exposure and is not cell-type-specific, as it also occurs in other cell lines of varied origin that are wild-type for *BRCA2* (Figure S3B). Neither HU nor a panel of other genotoxic agents (camptothecin [CPT], mitomycin C [MMC], ultraviolet light, ionizing radiation [IR], 5-azacytidine [5-Aza]) cause BRCA2 depletion in *BRCA2* wild-type cells even after extended exposure for up to 24 hr (Figure 3C) despite robust activation of the DNA damage response marked by increased phosphorylation of Ser 1981 in ataxia-telangiectasia-mutated (ATM) kinase and Thr 1989 in ATM-related (ATR) kinase. Thus, we unexpectedly find that formaldehyde depletes BRCA2 protein from many cell types that are wild-type for *BRCA2*, an effect not triggered by other genotoxins.

A pulse-chase assay using the protein synthesis inhibitor cycloheximide in *BRCA2* wild-type cells shows that 300 μM formaldehyde shortens the half-life of BRCA2 protein from 4.5 hr ± 1.7 to 2.3 hr ± 0.4 (Figure 3D). While three different inhibitors of the 20S proteasome (Kisselev et al., 2006)—velcade, epoxomicin, and MG132—substantially restored BRCA2 protein levels in formaldehyde-treated cells (Figure 3E), the lysosome inhibitor chloroquine (Solomon and Lee, 2009) did not. The deubiquitinase inhibitor, b-AP15 (that potently inhibits the recognition of polyubiquitinated substrates by the 19S proteasome) also fails to protect BRCA2 protein (Figure 3E), despite permitting the accumulation of many polyubiquitinated species. Several reports indicate that b-AP15 spares proteolysis by the 20S core proteasome (Chitta et al., 2015, Tian et al., 2014), but there is also evidence otherwise (Huang et al., 2014), precluding a definitive conclusion regarding the role of ubiquitination in the formaldehyde-induced proteasomal degradation of wild-type BRCA2 protein.

BRCA2 protein largely populates the nuclear-soluble fraction of cell extracts and is also detected in the chromatin-bound and cytosolic fractions (Figure S3C). Formaldehyde-induced BRCA2 degradation affects all three fractions, suggesting that the underlying mechanism operates in them all.

### Formaldehyde Selectively Depletes Components of the Cellular Proteome

**Figure 4:**
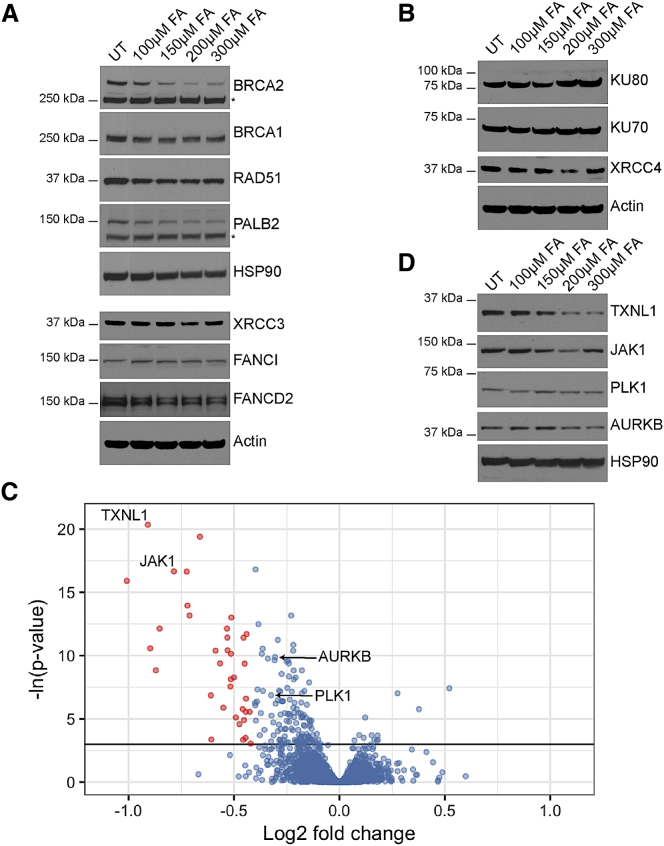
Formaldehyde Selectively Depletes Components of the Cellular Proteome (A) Abundance of proteins involved in homologous recombination or the Fanconi anemia repair pathway in wild-type HeLa Kyoto cells treated with FA for 5 hr. (B) Abundance of proteins involved in non-homologous end-joining in HeLa Kyoto cells treated with FA for 5 hr. (C) Volcano plot showing the results of the SWATH-MS analysis of HeLa Kyoto cells treated with or without 200 μM FA for 5 hr. Each dot represents a protein with Benjamini-Hochberg adjusted p values plotted along the y axis, and the fold change in abundance following FA treatment along the x axis. The horizontal black line indicates where p = 0.05. Red dots mark proteins that are depleted by ≥25% compared to untreated controls in a statistically significant manner (p < 0.05). Proteins tested by western blotting are labeled. (D) Abundance of selected proteins from SWATH-MS analysis in HeLa Kyoto cells treated with FA for 5 hr. See also Figure S4 and Tables S1 and S2.

Formaldehyde-induced degradation affects relatively few proteins encoded in the human proteome. Proteins other than BRCA2 implicated in homologous recombination or the Fanconi anemia repair pathway, like BRCA1, RAD51, PALB2, XRCC3, FANCI, or FANCD2, were at most modestly affected (Figure 4A), as were non-homologous end joining proteins like KU80, KU70, and XRCC4 (Figure 4B).

**Figure S4:**
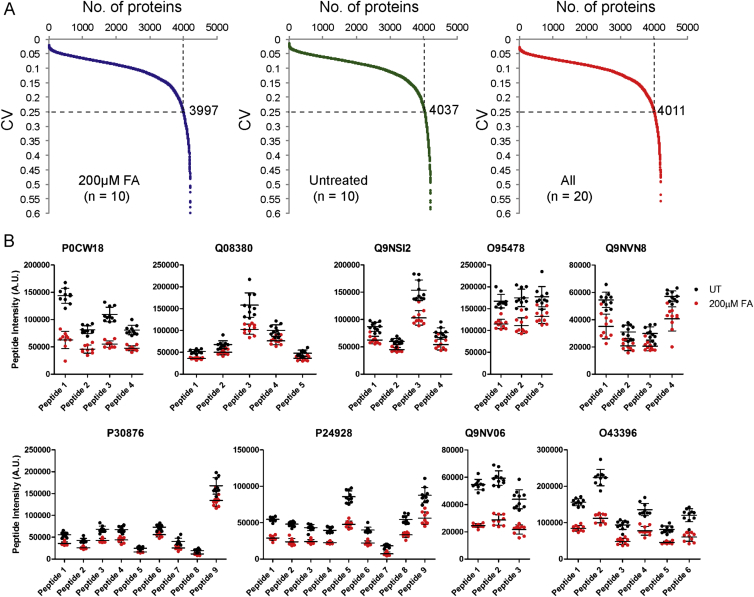
Related to Figure 4 (A) Coefficient of variation (CV) distribution of the normalized SWATH intensities for FA-treated samples (left panel, 10 replicates), untreated samples (middle panel, 10 replicates), and all samples (right panel, 10 + 10 samples). The line of CV at 25% is illustrated by the horizontal dotted line. (B) Normalized peptide intensities of individual peptides in at least 8 out of 10 biological replicates of 9 representative proteins showing statistically significant depletion of protein abundances by more than 25% after formaldehyde treatment.

The selectivity of protein depletion triggered by formaldehyde is evident in proteome-wide analyses using sequential window acquisition of all theoretical spectra-mass spectrometry (SWATH-MS), which quantifies protein abundance at high throughput and resolution (Gillet et al., 2012). In ten biological replicates per sample from HeLa Kyoto cells treated with or without 200 μM formaldehyde for 5 hr, we detected >50,000 peptides from all samples and analyzed only peptides detected in at least eight out of ten replicates in both conditions. The abundance of 4,219 proteins could be calculated by summating the peptide intensities of the three most abundant peptides for each protein. Analysis of fold-changes in protein expression and the associated Benjamini-Hochberg corrected p values (Table S1) reveals that <1% of the detected proteins (35/4,219) showed statistically significant reductions (p < 0.05, Benjamini-Hochberg adjusted) of more than 25% following formaldehyde exposure (Figure 4C; Table S2). Examples of the 35 depleted proteins (e.g., TXNL1 and JAK1) were robustly depleted by formaldehyde in western blotting, in contrast to proteins (e.g., PLK1 and AURKB) shown by SWATH-MS to exhibit little change in abundance, confirming complementarity between the two detection methods (Figure 4D). In addition, the number of proteins with coefficient of variation (CV) <25% was similar across all (treated and untreated) samples (Figure S4A). By contrast, for all of the 35 proteins affected by formaldehyde, peptide intensities were significantly and consistently decreased in at least eight out of ten biological replicates (nine representative examples plotted in Figure S4B). Thus, formaldehyde selectively reduces the abundance of a few additional proteins besides BRCA2, a previously unrecognized effect that may be salient to its role as a ubiquitous environmental toxin. However, features common to the proteins apparently targeted by formaldehyde are not yet evident, obscuring immediate insights into mechanism.

### Formaldehyde Induces Haploinsufficiency in *BRCA2* Heterozygous Cells, Causing Replication Stress

**Figure 5:**
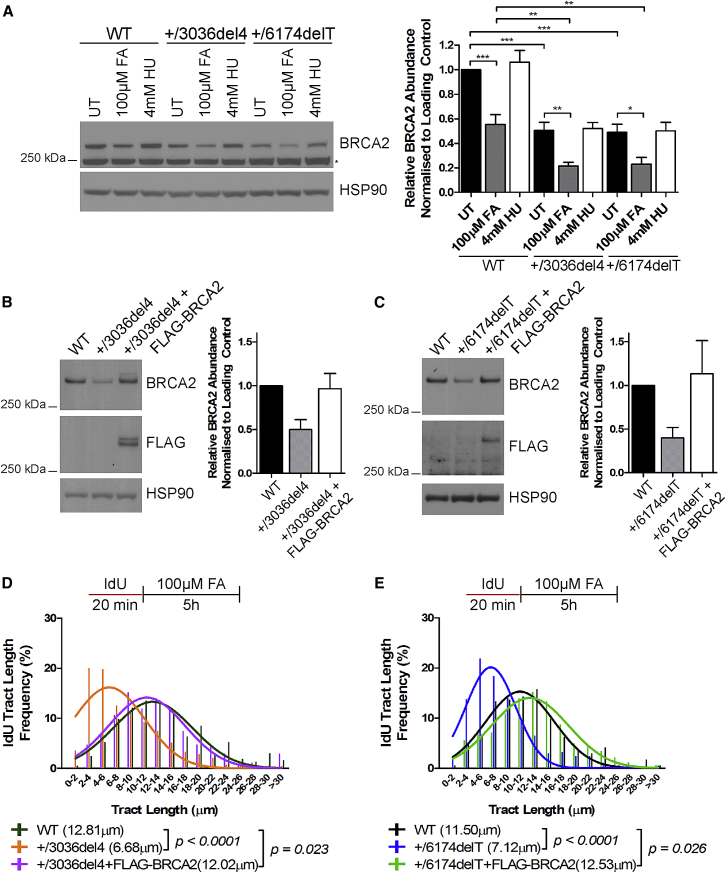
BRCA2 Complementation in *BRCA2* Heterozygous Cells Is Sufficient to Counteract Formaldehyde-Induced Replication Stress (A) BRCA2 abundance in HeLa Kyoto cells treated as indicated for 5 hr. Mean ± SEM of BRCA2 band intensities normalized to loading controls are plotted, n = 8. (B and C) BRCA2 abundance in (B) *BRCA2*^+/3036del4^ and (C) *BRCA2*^+/6174delT^ heterozygous cells complemented with FLAG-BRCA2, plotted as in (A). (D) IdU tract length frequency distributions of *BRCA2*^+/3036del4^ heterozygous cells complemented with FLAG-BRCA2 after FA exposure for 5 hr. (E) IdU tract length frequency distributions of *BRCA2*^+/6174delT^ heterozygous cells complemented with FLAG-BRCA2 after FA exposure for 5 hr. Results of D and E represent two independent experiments. See also Figure S5.

**Figure S5:**
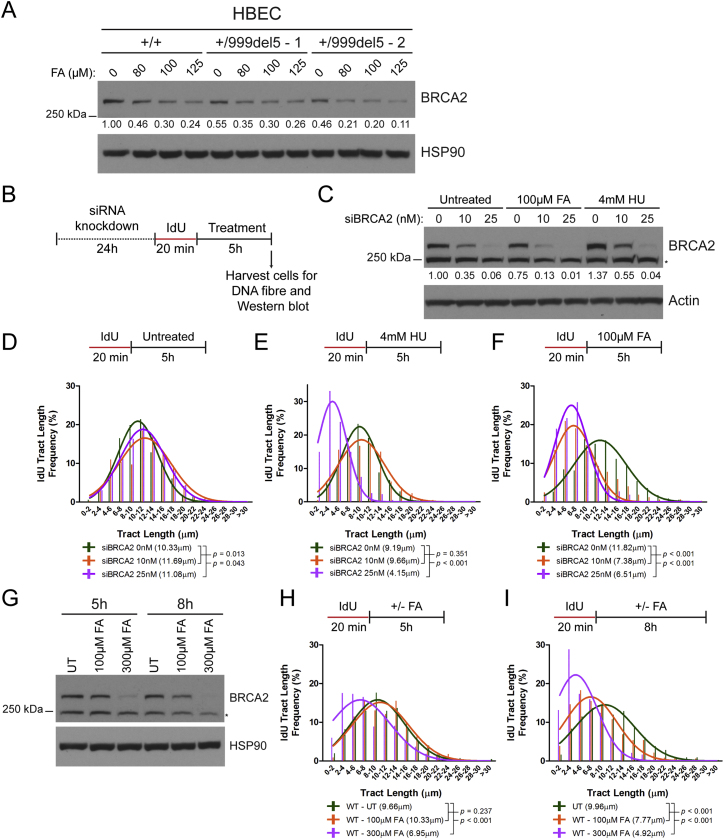
Related to Figure 5 (A) BRCA2 protein levels in *BRCA2*^+/+^ and *BRCA2*^+/999del5^ human breast epithelial cells (HBECs) treated with increasing doses of FA for 5h. Numbers below the BRCA2 blot represent densitometric measurements of BRCA2 protein levels normalized to loading control and untreated *BRCA2*^+/+^ cells (lane 1). Although *BRCA2*^+/999del5^ −1 HBECs have lower pre-existing levels of BRCA2 protein than their *BRCA2*^+/+^ counterparts, similar levels are reached after formaldehyde exposure. However, since *BRCA2*^+/999del5^ −1 HBECs exhibit replication tract instability after formaldehyde exposure whereas their *BRCA2*^+/+^ counterparts do not (Figure 2), these observations may reflect differences between these non-isogenic human cell lines in the kinetics of aldehyde-induced BRCA2 depletion, and/or in the level of BRCA2 that is adequate for function. (B) Experimental set-up for the siRNA knockdown DNA fiber experiment. (C) BRCA2 protein levels of wild-type HeLa Kyoto cells following transfection with BRCA2 short interfering RNA (siBRCA2) in combination with the indicated treatments. Numbers below the BRCA2 blot show the densitometric measurements of BRCA2 band intensities normalized to loading control and relative to the untreated control (lane 1). (D–F) IdU tract length frequency distributions of wild-type HeLa Kyoto cells treated under the indicated conditions. (G) BRCA2 protein levels in wild-type HeLa Kyoto cells after treatment with 100μM and 300μM FA for 5h and 8h. (H–I) IdU tract length frequency distributions in wild-type HeLa Kyoto cells after treatment with 100μM and 300μM FA for 5h and 8h respectively.

We next tested the effects of formaldehyde exposure on BRCA2 protein levels in *BRCA2* heterozygous cells, in which expression is already lowered to ∼50% of that in wild-type parental cells (Figure 2B). After exposure to 100 μM formaldehyde exposure for 5 hr, BRCA2 expression in parental cells decreased to ∼55% of the baseline value, approximately a 2-fold reduction. However, a similar 2-fold reduction induced by formaldehyde in cells heterozygous for the *BRCA2 6174delT* or *3036del4* mutations further diminished expression to ∼20% of wild-type levels (Figure 5A). In contrast, HU exposure had a minimal effect in all conditions. HBECs heterozygous for *BRCA2*^999del5^ responded similarly (Figure S5A).

Could formaldehyde exposure lead to “induced haploinsufficiency” for BRCA2 protein in settings where its expression is already lowered by heterozygous mutations, by reducing BRCA2 levels to a degree that compromises function? We complemented *BRCA2 6174delT* or *3036del4* heterozygous cells by stably expressing a construct encoding full-length FLAG epitope-tagged BRCA2 to restore wild-type levels of BRCA2 protein (Figures 5B and 5C). BRCA2 complementation sufficed to counteract formaldehyde-induced shortening of IdU-labeled replication tracts (Figures 5D and 5E). These results validate that formaldehyde indeed triggers “induced haploinsufficiency” for BRCA2 in cells bearing heterozygous mutations, and replication stress arises from the depletion of BRCA2 and not other proteins.

Conversely, we partially depleted BRCA2 protein from HeLa Kyoto parental cells using calibrated doses of siRNA before analyzing formaldehyde-induced replication stress (Figure S5B). Treatment with 10 nM versus 25 nM of siRNA progressively reduces BRCA2 protein expression from ∼35% to ∼6% of baseline levels in untreated cells (Figure S5C). When combined with 100 μM formaldehyde for 5 hr, BRCA2 levels are sharply further reduced by ∼3- to 6-fold, such that 10 nM of siRNA with formaldehyde reduces expression to ∼13% of baseline levels, and 25 nM of siRNA with formaldehyde reduces expression to ∼1% of baseline levels. In contrast, 4 mM HU combined with different doses of siRNA has little effect over siRNA alone.

BRCA2 depletion (from 35% to 6% of baseline levels) using siRNA alone had little effect on processive DNA replication in untreated cells (Figure S5D). BRCA2 depletion to 55% of baseline levels using 10 nM siRNA (mimicking *BRCA2* heterozygosity) did not alter the length of IdU-labeled replication tracts after replication stalling provoked by HU, whereas depletion to 4% of baseline levels using 25 nM siRNA (mimicking bi-allelic *BRCA2* inactivation) caused a marked degradation of the tracts (Figure S5E). In contrast, the combined effect of formaldehyde plus 10 nM or 25 nM siRNA reduced BRCA2 expression to 13% and 1% of baseline levels, respectively, triggering significant shortening of IdU-labeled replication tract length in both conditions (Figure S5F). Our findings provide additional evidence to support the idea that *BRCA2* haploinsufficiency potentiates formaldehyde-induced replication stress, suggesting that many of the clinically relevant frameshift, splicing, or nonsense mutations in *BRCA2* thus far detected in humans may have similar consequences.

In multiple experiments using different cell types, replication stress is not detected when cells wild-type for *BRCA2* are exposed to 80–100 μM formaldehyde for 3–5 hr, although it is evident in similarly treated *BRCA2* heterozygous cells (Figures 2E, 2H, 2I–2M, and S1C–S1H). Indeed, formaldehyde exposure in *BRCA2* wild-type cells reduces BRCA2 protein to ∼50% of pre-existing levels (Figure 3A, 5A, and S3A), consistent with the absence of replication stress. However, prolonged or high-dose formaldehyde treatment enhances BRCA2 protein depletion even in wild-type cells (Figure S5G), concomitantly precipitating replication tract instability (Figures S5H and S5I). This previously unrecognized mechanism may contribute to the genotoxicity of formaldehyde.

### Formaldehyde Triggers Structural Chromosome Aberrations in *BRCA2* Heterozygous Cells

**Figure 6:**
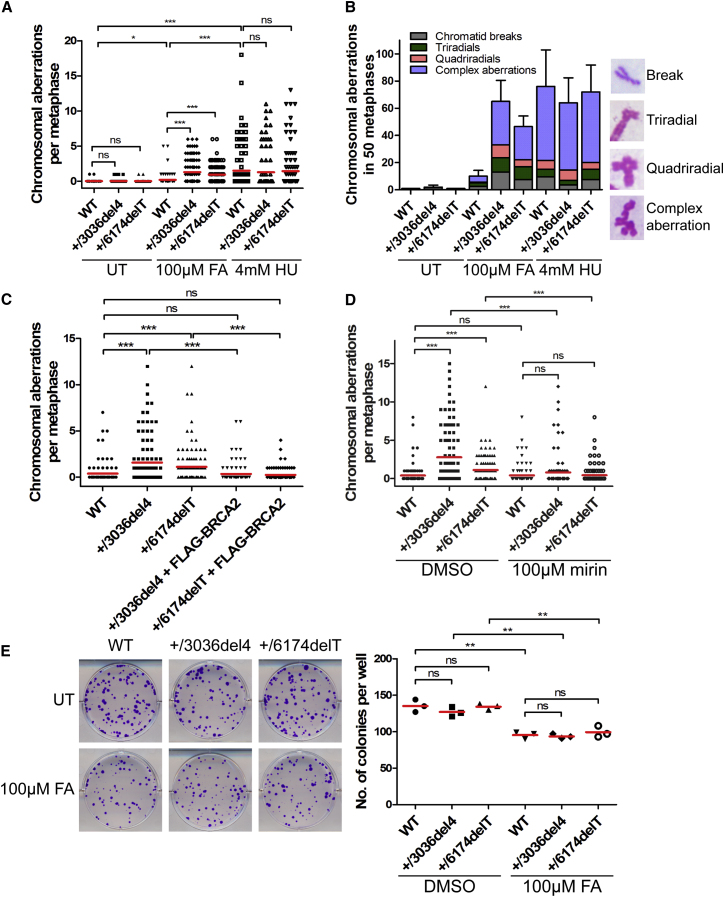
Formaldehyde Triggers Structural Chromosome Aberrations in *BRCA2* Heterozygous Cells (A) Frequency of chromosomal aberrations from HeLa Kyoto cells treated as indicated for 5 hr. Red lines indicate the mean, n = 2. (B) A breakdown of the different types of chromosomal aberrations observed in (A). Examples of various chromosomal aberrations are shown. (C) Frequency of chromosomal aberrations in HeLa Kyoto cells treated with 100 μM FA for 5 hr. Red lines indicate the mean, n = 2. (D) Frequency of chromosomal aberrations in HeLa Kyoto cells treated with 100 μM FA for 5 hr in the presence or absence of Mirin. Red lines indicate the mean, n = 2. (E) Representative images of colony formation by HeLa Kyoto cells treated with or without 100 μM FA for 5 hr. Each dot indicates the colony number per well. Red lines indicate the mean. Results represent two independent experiments. See also Figure S6.

**Figure S6:**
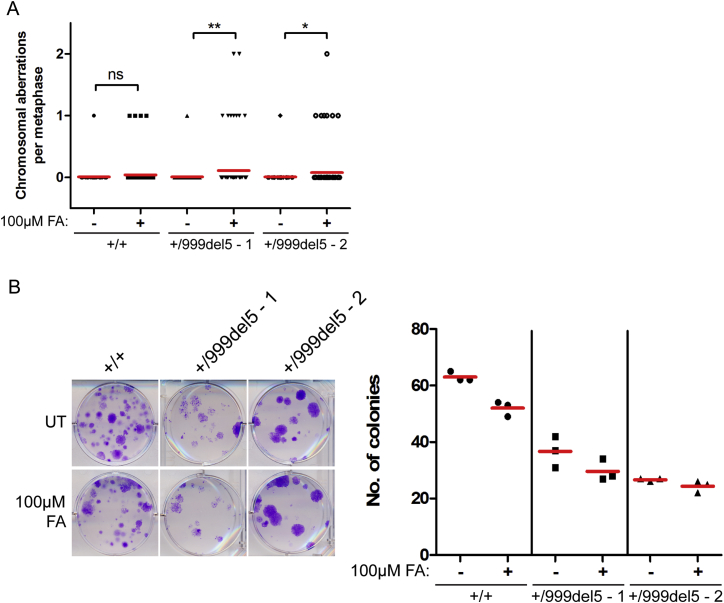
Related to Figure 6 (A) Frequency of chromosomal aberrations in metaphase spreads of HBECs treated with or without 100μM FA for 5h. Red lines indicate the mean, n = 2. (B) Representative images of a colony formation assay of human breast epithelial cells treated with or without 100μM FA for 5h. UT, untreated. The scatterplot shows the number of colonies per well from triplicate wells with red lines indicating the mean number of colonies per well. Representative of two independent experiments.

Replication fork stalling and instability have been linked to the genesis of chromosomal lesions through mechanisms that remain uncertain (Schlacher et al., 2011). Untreated cells heterozygous for the *BRCA2 6174delT* or *3036del4* mutations exhibit a low average frequency of structural chromosome aberrations equating to 0.02 ± 0.01 and 0.04 ± 0.02 per metaphase, respectively, similar to that observed in wild-type parental controls (0.02 ± 0.01 aberrations per metaphase) and consistent with previous findings (Patel et al., 1998). Strikingly, after treatment with formaldehyde, the average frequency of structural chromosomal aberrations per metaphase increased markedly—by over 30- to 40-fold (+/6174delT, 0.02 ± 0.01 to 0.93 ± 0.13; +/3036del4, 0.04 ± 0.02 to 1.30 ± 0.18)—in *BRCA2* heterozygous cells (Figures 6A and 6B). Similar results are observed using *BRCA2*^999del5^ HBECs (Figure S6A). Aberrations include Y-shaped tri-radial and star-shaped quadri-radial structures pathognomonic of defects in mitotic recombination that are characteristic of *BRCA2*-deficient cells (Patel et al., 1998, Yu et al., 2000). In contrast, the average frequency of chromosomal aberrations in parental cells exposed to formaldehyde did not change significantly (Figures 6A and 6B). Moreover, treatment with HU enhanced equally the frequency of chromosomal aberrations in both wild-type and *BRCA2* heterozygous cells. Complementation with full-length FLAG epitope-tagged BRCA2 of cells heterozygous for either the *BRCA2 6174delT* or *3036del4* mutations significantly reduced formaldehyde-induced chromosomal aberrations (Figure 6C), confirming that *BRCA2* haploinsufficiency accounts for this effect.

The MRE11 inhibitor, Mirin, reverses replication tract degradation triggered by formaldehyde in *BRCA2* heterozygous cells (Figures 2L and 2M). Mirin also significantly ameliorates the frequency of structural chromosomal aberrations in formaldehyde-treated *BRCA2* heterozygous cells (+/6174delT, 1.11 ± 0.18 to 0.44 ± 0.12; +/3036del4, 2.77 ± 0.38 to 0.80 ± 0.22) (Figure 6D), suggesting that nascent DNA resection at stalled replication forks contributes to chromosomal instability.

Formaldehyde-induced DNA replication stress and chromosomal aberrations in *BRCA2* heterozygous cells do not greatly impair clonogenic survival (Figure 6E). Formaldehyde exposure at 100 μM formaldehyde for 5 hr modestly reduced colony formation, as expected, but did so likewise in *BRCA2* heterozygous and wild-type parental cells. Similar results were observed using *BRCA2*^999del5^ HBECs (Figure S6B).

### Ribonuclease H1 Ameliorates Formaldehyde-Induced Replication Stress and Genome Damage

RNA-DNA hybrids (R-loops) are normal intermediates in DNA transactions such as transcription termination, but their unscheduled formation may trigger genomic instability (Hatchi et al., 2015, Huertas and Aguilera, 2003). Interestingly, unscheduled R-loops accumulate after bi-allelic inactivation of *BRCA2* (Bhatia et al., 2014) and may contribute to replication stress in cells that lack FANCD2 or FANCA (García-Rubio et al., 2015, Schwab et al., 2015). To test whether R-loops mediate formaldehyde-induced replication stress and genome damage, we used ribonuclease (RNase) H1, whose overexpression efficiently dissolves R-loops (Cerritelli and Crouch, 2009) and has previously been deployed to test their involvement in cellular processes (Bhatia et al., 2014, Schwab et al., 2015).

**Figure 7:**
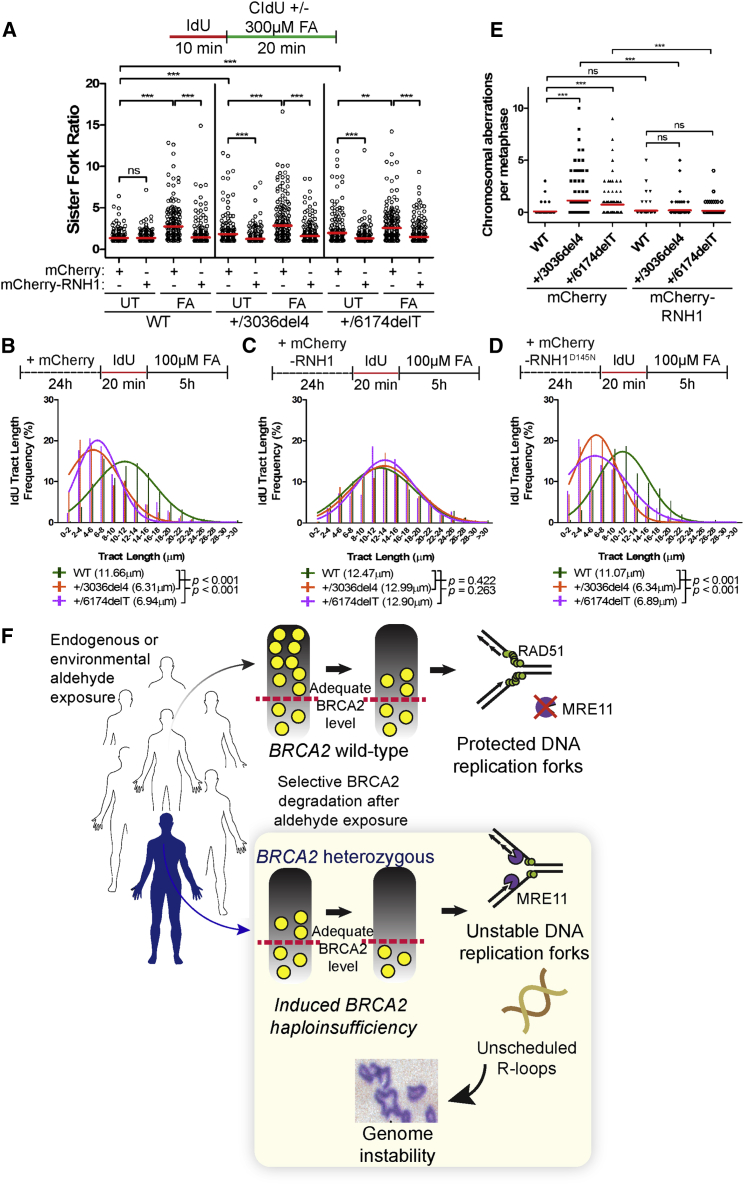
Ribonuclease H1 Ameliorates Formaldehyde-Induced Replication Stress and Genome Damage (A) DNA fiber assay comparing sister fork symmetry in HeLa Kyoto cells expressing mCherry or mCherry-RNase H1 (RNH1) vectors with or without FA treatment. The scatterplot compares the ratio of sister-fork tract lengths between the different conditions with red lines indicating the median, n = 2. (B) IdU tract length frequency distributions of HeLa Kyoto cells transiently expressing mCherry after FA exposure for 5h. (C) IdU tract length frequency distributions of HeLa Kyoto cells transiently expressing mCherry-RNase H1 after FA exposure for 5h. (D) IdU tract length frequency distributions of HeLa Kyoto cells transiently expressing mCherry-RNase H1 (D145N) after FA exposure for 5h. Results of (B)–(D) represent at least two independent experiments. (E) Frequency of chromosomal aberrations in HeLa Kyoto cells expressing either mCherry or mCherry-RNase H1 following 5 hr treatment with 100 μM FA. Red lines indicate the mean, n = 2. (F) Aldehyde-induced haploinsufficiency in *BRCA2* heterozygous cells. Aldehyde exposure triggers selective BRCA2 degradation in the cells of both wild-type individuals as well as those who carry heterozygous *BRCA2* mutations. Adequate levels of BRCA2 remain in wild-type individuals. But in *BRCA2* heterozygous mutation carriers, aldehyde-induced degradation decreases BRCA2 levels below the threshold of adequacy, engendering “induced haploinsufficiency.” These events expose stalled DNA replication forks to MRE11 activity, engendering chromosomal aberrations via R-loop formation. See also Figure S7.

**Figure S7:**
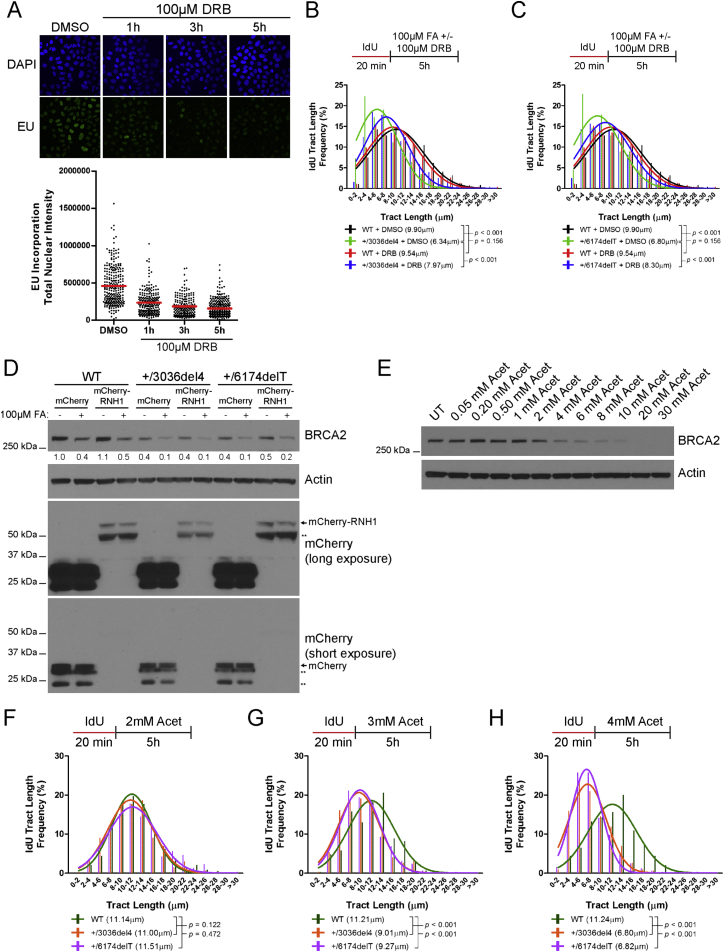
Related to Figure 7 (A) Immunofluorescence images of wild-type HeLa Kyoto cells treated with the transcription inhibitor, 5,6-dichloro-1-β-D-ribofuranosylbenzimidazole (DRB) at 100μM for the indicated lengths of time and subsequently labeled with EU for 1h as measure of total RNA synthesis. The scatterplot shows the total nuclear intensities of EU signal from at least 200 nuclei per condition with red lines indicating the median and each dot representing a single nucleus. (B and C) IdU tract length frequency distributions in HeLa Kyoto cells after concurrent treatment with 100μM FA and 100μM DRB for 5h. (D) BRCA2 protein levels in HeLa Kyoto cells expressing mCherry or mCherry-RNase H1 vectors after exposure to 100μM FA for 5h. Numbers below the BRCA2 blot represent densitometric measurements of BRCA2 protein levels normalized to loading control and lane 1 of the BRCA2 blot. Different exposures of mCherry blots show expression of mCherry and mCherry-RNase H1 as indicated. Double asterisks (^∗∗^) indicate probable degradation products. (E) BRCA2 protein levels in wild-type HeLa Kyoto cells after treatment with increasing doses of acetaldehyde for 5h. (F–H) IdU tract length frequency distributions in HeLa Kyoto cells after treatment with 2, 3 or 4 mM acetaldehyde for 5h.

Strikingly, there is a marked decrease in the ratio of sister fork tract lengths in formaldehyde-exposed cells expressing an mCherry fluorophore-tagged form of RNase H1, but not mCherry alone (Figure 7A), suggesting that R-loops significantly contribute to formaldehyde-induced replication stalling in parental as well as *BRCA2* heterozygous cells. *BRCA2 6174delT* or *3036del4* heterozygous cells exhibit—even when untreated—a modest elevation in the ratio of sister fork tract lengths that is reduced by overexpression of mCherry-RNase H1, but not mCherry alone (Figure 7A). We infer that *BRCA2* heterozygous cells experience increased levels of replication fork stalling due to R-loops even during unperturbed replication in culture, although this defect does not per se augment chromosomal aberrations (Figures 6A and 6B). Furthermore, transcription inhibition by 5,6-dichloro-1-β-D-ribofuranosylbenzimidazole (DRB) (Figures S7A–S7C) or the overexpression of mCherry-RNase H1—but neither mCherry alone nor the inactive Asp145Asn (D145N) mutant of mCherry-RNase H1 (compare Figures 7B–7D)—also reduce replication tract shortening in formaldehyde-treated *BRCA2* heterozygous cells. Moreover, formaldehyde-induced BRCA2 degradation persists despite mCherry-RNase H1 overexpression (Figure S7D), suggesting it is a cause, not the consequence, of excessive R-loops. Thus, multiple lines of evidence implicate unscheduled R-loop formation in the genesis of formaldehyde-induced replication stress in *BRCA2* heterozygous cells.

Remarkably, overexpression of mCherry-RNase H1, but not mCherry alone, significantly decreases the frequency of structural chromosomal aberrations in *BRCA2* heterozygous cells after formaldehyde exposure to levels comparable to wild-type parental cells (Figure 7E). Thus, remarkably, our data suggest that R-loop formation not only underlies formaldehyde-induced replication fork stalling but also instigates genomic instability in *BRCA2* heterozygous cells.

### Acetaldehyde-Induced Replication Tract Shortening in *BRCA2* Heterozygous Cells

Acetaldehyde, an endogenous product of ethanol catabolism, is detoxified by ALDH2, whose activity is lost in >500 million people worldwide through an inherited, *trans*-dominant mutation, *ALDH2*^*E487K*^, prevalent in individuals of East Asian descent (Yoshida et al., 1984). Carriers of this mutation typically exhibit adverse reactions to alcohol consumption, due to the build-up of acetaldehyde. Interestingly, transient exposure to acetaldehyde induces BRCA2 protein degradation in a dose-dependent manner (Figure S7E), and DNA fiber analysis confirms that IdU-labeled replication tracts are significantly shortened in *BRCA2 6174delT* or *3036del4* heterozygous, but not parental cells after treatment with 3–4 mM acetaldehyde for 5 hr (Figures S7F–S7H). These findings suggest that the mechanism of “induced *BRCA2* haploinsufficiency” may be common to different aldehydes.

## Discussion

We report here that naturally occurring concentrations of formaldehyde, a product of cellular metabolism and a ubiquitous environmental toxin, provoke replication fork instability and structural chromosomal aberrations in cells heterozygous for multiple, pathogenic truncating mutations affecting the *BRCA2* tumor suppressor. These anomalies arise from a previously unrecognized effect of formaldehyde to selectively deplete BRCA2 via proteasomal degradation. Settings where *BRCA2* expression is already compromised by heterozygous truncating mutations potentiate vulnerability to formaldehyde-induced haploinsufficiency for this tumor suppressor protein. Similar effects occur with acetaldehyde, a product of ethanol catabolism. We propose a model (Figure 7F) wherein aldehyde exposure potentiates the carcinogenic potential of germline truncating mutations affecting a single allele of *BRCA2*. Our findings have several implications.

### Mechanism of Formaldehyde-Induced Chromosomal Instability in *BRCA2* Heterozygous Cells

We provide a first line of evidence that replication fork degradation by MRE11 contributes to chromosomal instability (Figures 2L, 2M, and 6D). MRE11 inhibitors significantly reduce structural chromosomal aberrations induced by formaldehyde in *BRCA2 6174delT* or *3036del4* heterozygous cells, suggesting that two different effects of formaldehyde—DNA replication fork stalling (Figures 1B, 2, and S2) plus induced haploinsufficiency for BRCA2 (Figure 5)—collude to precipitate chromosomal instability.

We reveal a previously unrecognized link between unscheduled RNA-DNA hybrid formation and formaldehyde-induced chromosomal instability in *BRCA2* heterozygous cells. Overexpression of RNase H1, an enzyme that resolves R-loops, ameliorates formaldehyde-induced replication fork instability (Figures 7B–7D) and chromosomal aberrations (Figure 7E) in *BRCA2* heterozygous cells. Notably, RNase H1 mitigates chromosomal aberrations thought typical of defective mitotic recombination, and thus currently accepted models attributing chromosomal instability solely to defective homologous DNA recombination will need to be extended.

### Selective Proteome Depletion Induced by Formaldehyde

Unexpectedly, formaldehyde selectively depletes the cellular proteome, an effect that is neither cell-type-specific nor induced by several other forms of DNA damage (Figures 3C and S3B). Besides BRCA2, only ∼35 of >4,200 proteins detected by SWATH-MS are selectively depleted. The mechanism(s) underlying the selectivity of formaldehyde-induced proteome depletion remain unclear and warrant further study. However, this first systematic analysis of the effect of formaldehyde on proteome expression identifies changes that may be linked in future studies to the toxicity of this ubiquitous, reactive compound.

### Implications for Carcinogenesis and Public Health

Our findings suggest a new model (Figure 7F) for carcinogenesis in individuals who carry germline mutations truncating a single copy of *BRCA2*. Exposure to aldehydes like formaldehyde or acetaldehyde, which are both widespread in our environment and also accumulate endogenously in certain tissues, could potentiate spontaneous mutagenesis in the cells of mutation carriers, predisposing to cancer. In this model, the risk of carcinogenesis among mutation carriers may depend not only on the nature of their germline *BRCA2* mutation and its susceptibility to aldehyde-induced haploinsufficiency but also upon other genetic and environmental factors that determine exposure to aldehydes. Because *BRCA2* mutation carriers typically develop cancers in certain epithelial tissues including the breast, ovary, pancreas, or prostate, we speculate that organ-specific differences in endogenous aldehyde accumulation or extrinsic exposure may account in part for the observed tissue selectivity.

Notably, our proposal suggests that—rather than being promoted solely by intrinsic cellular defects—cancer evolution among carriers of at least certain types of *BRCA2* mutations may instead be driven by a gene-environment interaction, in which a category of pervasive, naturally occurring compounds trigger “induced haploinsufficiency.”

Even in wild-type cells, prolonged or high-dose formaldehyde exposure can deplete BRCA2 protein to levels low enough to induce replication stress (Figures S5G–S5I). Thus, although *BRCA2* heterozygous cells are particularly vulnerable to aldehyde-induced haploinsufficiency, wild-type cells are not impervious, suggesting a mechanism for the carcinogenic potential of formaldehyde exposure in otherwise normal individuals.

The public health significance of our findings is emphasized by the ubiquity of exposure to formaldehyde and acetaldehyde, particularly in the urban environment, from sources including tobacco smoke, e-cigarettes, automobile combustion emissions, building materials, and even cosmetics (IARC, 2006). Over 500 million individuals worldwide, particularly of East Asian descent, carry the *trans*-dominant *ALDH2*^*E487K*^ polymorphic variant, which vastly reduces enzymatic activity for acetaldehyde catabolism leading to increased acetaldehyde build-up after alcohol consumption (Jin et al., 2015). Epidemiological studies seem warranted to investigate the risk of cancer associated with *BRCA2* mutations in such populations, particularly in light of the difficulty in testing these hypotheses in genetically engineered pre-clinical mouse models. Conversely, it is tempting to speculate that dietary supplementation with formaldehyde scavengers like Resveratrol (Marcsek et al., 2007) may offer a future strategy to reduce cancer incidence in patients who carry pathogenic truncating mutations affecting *BRCA2*.

## STAR★Methods

### Key Resources Table

**Table:** 

REAGENT or RESOURCE	SOURCE	IDENTIFIER
**Antibodies**		

Rat anti-BrdU	Abcam	Cat#ab6326; RRID: AB_305426
Chicken anti-rat AlexaFluor 488	ThermoFisher Scientific	Cat#A-21470; RRID: AB_2535873
Goat anti-chicken AlexaFluor 488	ThermoFisher Scientific	Cat#A-11039; RRID: AB_142924
Mouse anti-BrdU	BD Biosciences	Cat#347580; RRID: AB_10015219
Rabbit anti-mouse AlexaFluor 594	ThermoFisher Scientific	Cat#A-11062; RRID: AB_2534109
Goat anti-rabbit AlexaFluor 594	ThermoFisher Scientific	Cat#A-11012; RRID: AB_141359
Anti-single strand DNA	Merck Millipore	Cat#MAB3034; RRID: AB_94645
Rabbit anti-mouse AlexaFluor 350	ThermoFisher Scientific	Cat#A-21062; RRID: AB_2535726
Goat anti-rabbit AlexaFluor 350	ThermoFisher Scientific	Cat#A-11046; RRID: AB_142716
Rabbit anti-mouse AlexaFluor 488	ThermoFisher Scientific	Cat#A-11059; RRID: AB_142495
Goat anti-rabbit AlexaFluor 488	ThermoFisher Scientific	Cat#A-11034; RRID: AB_2576217
Rabbit polyclonal anti-PCNA	Abcam	Cat#ab18197; RRID: AB_2576217
Mouse monoclonal anti-γH2AX	Merck Millipore	Cat#05–636; RRID: AB_309864
Mouse polyclonal anti-RAD51 (Immunofluorescence)	Abnova	Cat# H00005888-B01P; RRID: AB_1579507
Mouse monoclonal anti-BRCA2 (Ab-1)	Merck Millipore	Cat#OP95; RRID: AB_2067762
Rabbit polyclonal anti-BRCA1 (C-20)	Santa Cruz Biotechnology	Cat#sc-642; RRID: AB_630944
Rabbit polyclonal anti-PALB2	Bethyl Laboratories	Cat#A301-246A; RRID: AB_890607
Mouse monoclonal anti-RAD51 (western blot)	GeneTex	Cat#GTX70230; RRID: AB_372856
Rabbit monoclonal anti-phospho-ATM (S1981)	Abcam	Cat#ab81292; RRID: AB_1640207
Mouse monoclonal anti-ATM	Sigma-Aldrich	Cat#A1106; RRID: AB_796190
Rabbit polyclonal anti-phospho-ATR (T1989)	GeneTex	Cat#GTX128145
Rabbit polyclonal anti-ATR	Bethyl Laboratories	Cat#A300-137A; RRID: AB_185544
Rabbit monoclonal anti-LC3B	Cell Signaling	Cat#3868; RRID: AB_2137707
Mouse monoclonal anti-Flag	Sigma-Aldrich	Cat#F1804; RRID: AB_262044
Mouse monoclonal anti-mono- and polyubiquitinylated conjugates monoclonal antibody	Enzo Life Sciences	Cat#BML-PW8810; RRID: AB_10541840
Rabbit monoclonal anti-Hsp90	Cell Signaling	Cat#4877; RRID: AB_10829038
Mouse monoclonal anti-beta-actin	Sigma-Aldrich	Cat#A5441; RRID: AB_476744
Rabbit polyclonal anti-XRCC3	Merck Millipore	Cat#PC691; RRID: AB_2304699
Rabbit polyclonal anti-FANCI	Abcam	Cat#ab15344; RRID: AB_443182
Goat polyclonal anti-Ku80	Santa Cruz Biotechnology	Cat#sc-1485; RRID: AB_2288756
Mouse monoclonal anti-Ku70	Abcam	Cat#ab3114; RRID: AB_2219041
Rabbit polyclonal anti-XRCC4	Abcam	Cat#ab145; RRID: AB_301278
Mouse monoclonal anti-mCherry	Novus Biologicals	Cat#NBP1-96752; RRID: AB_11034849
Rabbit polyclonal anti-Histone H3	Cell Signaling	Cat#9715; RRID: AB_331563
Mouse monoclonal anti-c-Myc	Santa Cruz Biotechnology	Cat#sc-40; RRID: AB_627268
Rabbit monoclonal anti-TXNL1	Abcam	Cat#ab188328
Rabbit polyclonal anti-Aurora B	Abcam	Cat#ab2254; RRID: AB_302923
Mouse monoclonal anti-PLK1	ThermoFisher Scientific	Cat#33-1700; RRID: AB_2533104
Mouse monoclonal anti-JAK1	Santa Cruz Biotechnology	Cat#sc-376996

**Chemicals, Peptides, and Recombinant Proteins**		

37% formaldehyde solution	Sigma-Aldrich	Cat#F8875
Acetaldehyde solution	Sigma-Aldrich	Cat#402788
Hydroxyurea	Sigma-Aldrich	Cat#H8627
Mirin	Sigma-Aldrich	Cat#M9948
Epoxomicin	Biovision	Cat#2190-50
b-AP15	Cayman Chemical	Cat#CAY11324
Velcade	Selleckchem	Cat#S1013
Chloroquine	Sigma-Aldrich	Cat#C6628
MG132	Merck Millipore	Cat#474790
Mitomycin C	Sigma-Aldrich	Cat#M4287
Camptothecin	Sigma-Aldrich	Cat#C9911
5-azacytidine	Sigma-Aldrich	Cat#A3656
Cycloheximide	Sigma-Aldrich	Cat#C4859
Colcemid	ThermoFisher Scientific	Cat#15212012
Giemsa Stain solution	ThermoFisher Scientific	Cat#10092013
Gurr’s Buffer pH 6.8	ThermoFisher Scientific	Cat#10582013
Permount Mounting Medium	VWR	Cat#100496-550
Crystal Violet Solution	Sigma-Aldrich	Cat#HT90132
5-Iodo-2-deoxyuridine	Sigma-Aldrich	Cat# I7125
5-Chloro-2-deoxyuridine	Sigma-Aldrich	Cat#C6891
Blocking reagent for nucleic acid hybridization and detection	Roche	Cat#11096176001
Human insulin	Sigma-Aldrich	Cat#I9278
apo-Transferrin	Sigma-Aldrich	Cat#T1147
Epidermal growth factor	Peprotech	Cat#AF-100-15
Sodium selenite	Sigma-Aldrich	Cat#S5261
β-estradiol	Sigma-Aldrich	Cat#E2758
Hydrocortisone	Sigma-Aldrich	Cat#H0888
Prolactin	Sigma-Aldrich	Cat#L6520
5,6-Dichlorobenzimidazole 1-β-D-ribofuranoside	Sigma-Aldrich	Cat#D1916
JetPRIME Transfection Reagent	Polyplus Transfections	Cat#114-07
cOmplete, EDTA-free Protease Inhibitor Cocktail	Roche	Cat#11873580001

**Critical Commercial Assays**		

Click-iT EdU Alexa Fluor® 488 Imaging Kit	ThermoFisher Scientific	Cat#C10086
Click-iT RNA Alexa Fluor® 594 Imaging Kit	ThermoFisher Scientific	Cat#C10330
Subcellular Protein Fractionation Kit for Cultured Cells	ThermoFisher Scientific	Cat#78840

**Deposited Data**		

SWATH-MS proteomics data have been deposited to the ProteomeXchange Consortium via the PRIDE partner repository	This paper	ProteomeXchange: PXD006106

**Experimental Models: Cell Lines**		

Human: HeLa Kyoto	From the laboratory of Jonathan Pines	N/A
Human: HeLa Kyoto *BRCA2* +/3036del4	This paper	N/A
Human: HeLa Kyoto *BRCA2* +/6174delT	This paper	N/A
Human: HeLa Kyoto *BRCA2* +/3036del4 complemented with *FLAG-BRCA2*	This paper	N/A
Human: HeLa Kyoto *BRCA2* +/6174delT complemented with *FLAG-BRCA2*	This paper	N/A
Human: EUFA423	VU University Medical Center	N/A
Human: EUFA423 complemented with *FLAG-BRCA2*	Hattori et al., 2011	N/A
Human: U2OS	ATCC	Cat#HTB-96
Human: MCF-10A	ATCC	Cat#CRL-10317
Human: MCF7	ATCC	Cat#HTB-22
Human: hTERT-RPE1	ATCC	Cat#CRL-4000
Human: Breast epithelial cells *BRCA2* +/+	Rubner Fridriksdottir et al., 2005	N/A
Human: Breast epithelial cells *BRCA2* +/999del5 - 1	Rubner Fridriksdottir et al., 2005	N/A
Human: Breast epithelial cells *BRCA2* +/999del5 - 2	Rubner Fridriksdottir et al., 2005	N/A

**Recombinant DNA**		

Plasmid: pcDNA3.1(-) mCherry	This paper	N/A
Plasmid: pcDNA3.1(-) mCherry-RNase H1	This paper	N/A
Plasmid: pcDNA3.1(-) mCherry-RNase H1 (D145N)	This paper	N/A

**Sequence-Based Reagents**		

siRNA: siLuciferace: 5′-CGUACGCGGAAUACUUCGA-3′	This paper	N/A
siRNA: siBRCA2: 5′-GAAGAAUGCAGGUUUAAUA-3′	This paper	N/A

**Software and Algorithms**		

Cellomics Bioapplication Compartmental Analysis V4 Version 6.0	ThermoFisher Scientific	https://www.thermofisher.com/us/en/home/life-science/cell-analysis/cellular-imaging/high-content-screening/high-content-screening-instruments/hcs-studio-2.html
IncuCyte Version 2011A	Essen Bioscience	http://www.essenbioscience.com/en/products/software/incucyte-base-software/
Prism 5	Graphpad software	https://www.graphpad.com/scientific-software/prism/; RRID: SCR_002798
R statistical package Version 3.3.1	R Core Team	https://www.r-project.org/; RRID: SCR_001905
OpenSWATH	Röst et al., 2014;	http://www.openswath.org
Image Studio Lite Version 3.1	LI-COR Biosciences	https://www.licor.com/bio/products/software/image_studio/; RRID: SCR_013715
ImageJ Version 1.47 m	Wayne Rasband (NIH)	https://imagej.nih.gov/ij

### Contact for Reagent and Resource Sharing

Further information and requests for resources and reagents should be directed to Lead Contact Ashok R. Venkitaraman (arv22@mrc-cu.cam.ac.uk).

### Experimental Model and Subject Details

#### Cell lines

HeLa Kyoto, EUFA423, U2OS and MCF7 cells (all female in gender) were cultured in DMEM supplemented with 10% FCS and 1% Penicillin/Streptomycin. hTERT-RPE1 cells were cultured in DMEM/F12 supplemented with 10% FCS, 1% Penicillin/Streptomycin and 4.2% sodium bicarbonate. MCF-10A cells were cultured in DMEM/F12 supplemented with 5% horse serum, 10 μg/ml insulin, 20ng/ml epidermal growth factor (EGF), 100ng/ml choleratoxin, 500ng/ml hydrocortisone and 1% penicillin/streptomycin. Human female breast epithelial cells were cultured in DMEM/F12 supplemented with 250ng/ml human insulin, 10μg/ml apo-transferrin, 10ng/ml EGF, 2.6ng/ml sodium selenite, 0.1nM β-estradiol, 0.5μg/ml hydrocortisone, 5μg/ml prolactin and 1% penicillin/streptomycin. Cells were grown on plastic dishes and maintained at 37 °C with 5% CO_2_. HeLa Kyoto *BRCA2* +/3036del4 and +/6174delT heterozygous cells were engineered using CRISPR/Cas9 technology and authenticated by DNA sequencing and western blotting to observe reduced levels of full-length BRCA2 protein.

### Method Details

#### EdU and EU Incorporation

For EdU labeling, cells were labeled with 10 μM EdU for 1h and fixed with 4% paraformaldehyde for 10 min at room temperature. For EU labeling, cells were labeled with 1mM EU for 1h and fixed with 4% paraformaldehyde for 10 min at room temperature. See the ‘*Immunofluorescence’* section for immunostaining procedures.

#### Immunofluorescence

For detection of chromatin-bound PCNA, cells grown on coverslips were pre-extracted with CSK buffer (25mM HEPES, pH 7.4, 50mM NaCl, 1mM EDTA, 3mM MgCl_2_, 300mM sucrose, 0.5% Triton X-100) for 5 min on ice, washed twice in 1x PBS and fixed in 4% paraformaldehyde solution for 10 min at 25°C. For EdU detection, we used the Click-iT reaction cocktail (Click-iT EdU Alexa Fluor 488 Imaging Kit, ThermoFisher, C10086) and applied onto samples for 30 min at room temperature, protected from light. After washing twice with 1X PBS, samples were blocked with 2% BSA, 0.1% Triton X-100/1X TBS solution for 30min and incubated with primary antibodies at 25°C for 2h (anti-γH2AX (Millipore #05–636, 1:2000), anti-PCNA, Abcam ab18197, 1:1000, anti-RAD51 (Abnova #H00005888-B01P, 1:2000)). Cells were washed thrice in 0.1% Triton X-100/1X TBS solution and incubated with appropriate secondary antibodies conjugated to Alexa fluorophores (Molecular Probes, 1:500) for 1h at 25°C. After three washes in 0.1% Triton X-100/1X TBS, slides were mounted with Vectashield containing 4’,6-diamidino-2-phenylindole (DAPI) and imaged using a Leica SP5 confocal microscope using 40x or 63x objective lenses.

For EU detection, fixed cells were washed twice with 1X PBS. After permeabilization with 0.5% Triton X-100/1X PBS for 20min at room temperature, the Click-iT reaction cocktail (Click-iT RNA Alexa Fluor 594 Imaging Kit, ThermoFisher C10330) was applied to samples for 30min at room temperature protected from light. After washing twice in 1X PBS, samples were mounted with Vectashield containing DAPI and imaged using a Leica SP5 confocal microscope using a 63x objective lens. See the ‘*Image acquisition and analysis’* section for details.

#### DNA Fiber Assay

For sister fork asymmetry assays, cells were labeled with IdU (25 μM) for 10min and subsequently with CIdU (250 μM) with or without formaldehyde for 20min. For replication fork stability assays, cells were labeled with IdU (25 μM) for 20min prior to incubation for 5h in respective treatments. Cells were spotted onto glass slides and lysed (200mM Tris-HCl, pH 7.4, 50mM EDTA, 0.5% SDS). DNA was combed by tilting of slides, air-dried and fixed in Carnoy’s fixative (10min, 25°C). Slides were dried and denatured in 2.5M HCl for 1h before washing 3x in ice-cold 1X PBS. Slides were blocked in 1.5% blocking solution (Roche, 11096176001, 0.05% Tween, 1X PBS, pH 7.4) for 30min at 37°C or overnight at 4°C. To detect CIdU, slides were incubated (45min, 25°C) with rat anti-BrdU (ab6326, 1:750) before incubated in stringency buffer (10mM Tris-HCl pH 7.4, 400mM NaCl, 0.2% Tween, 0.2% NP-40) for 15min at 25°C. Slides were washed thrice in 1X PBS and sequentially stained with secondary antibody (chicken anti-rat AF488, 1:200, 20min, 25°C) and tertiary antibody (goat anti-chicken AF488, 1:200, 20min, 25°C) with three PBS washes in between each antibody incubation. To detect IdU, slides were incubated (45min, 25°C) with mouse anti-BrdU (BD #347580, 1:5) and sequentially with secondary antibody (rabbit anti-mouse AF594, 1:50, 20min, 25°C) and tertiary antibody (goat anti-rabbit AF594, 1:50, 20min, 25°C) with three PBS washes in between each antibody incubation. Single-stranded DNA was stained (45min, 25°C) with mouse anti-ssDNA (MAB3034, Merck Millipore, 1:50) and sequentially with secondary antibody (rabbit anti-mouse AF488 or AF350, 1:50, 20min, 25°C) and tertiary antibody (goat anti-rabbit AF488 or AF350, 1:50, 20min, 25°C) with three PBS washes between each antibody incubation. Slides were mounted in 90% glycerol in 1X PBS and imaged using Leica SP5 confocal microscope. Tract lengths were measured using ImageJ.

#### Plasmid and siRNA transfections

JetPRIME transfection reagent (Polyplus Transfection, 114-07) was used for all plasmid and siRNA transfections, with 1μg of plasmid DNA per well of a 6-well dish. A 1:2 ratio of plasmid DNA (μg): JetPRIME reagent (μl) was used. Transfection reaction mixtures were vortexed thoroughly and incubated at room temperature for 10min before adding dropwise to cells. For siRNA transfections, 4μl of JetPRIME reagent was used per well of a 6-well dish. Transfection reaction mixtures were vortexed thoroughly and incubated at room temperature for 15min before adding dropwise to cells. Culture media was replaced with fresh media after 5h incubation at 37°C. siRNA sequences used: siLuciferase: 5′-CGUACGCGGAAUACUUCGA-3′; siBRCA2: 5′-GAAGAAUGCAGGUUUAAUA-3′.

#### Subcellular Fractionation

2.5 × 10^6^ HeLa Kyoto cells were seeded on 10 cm dishes. 24h after seeding, cells were treated and harvested by trypsinisation after 3h. Cell aliquots were taken from each sample for making whole cell extracts using RIPA buffer for lysis. The rest of the cells were lysed and fractionated using the Subcellular Protein Fractionation Kit for Cultured Cells (ThermoFisher Scientific, #78840). Briefly, cytoplasmic extraction buffer was added to cell pellets and samples were incubated at 4°C with gentle mixing for 10min. After centrifuging at 500 g for 5min, the supernatants were transferred to fresh tubes (cytoplasmic fraction). Next, membrane extraction buffer was added to pellets, vortexed, and incubated at 4°C with gentle mixing for 10min. After centrifuging at 3000 g for 5min, the supernatants were transferred to fresh tubes (membrane-bound fraction). Next, nuclear extraction buffer was added to pellets, vortexed thoroughly, and incubated at 4°C with gentle mixing for 30min. After centrifuging at 5000 g for 5min, the supernatants were transferred to fresh tubes (nuclear soluble fraction). Finally, chromatin-bound extraction buffer (prepared according to kit instructions) was added to remaining pellets, which were thoroughly resuspended by pipetting. After incubation at room temperature for 15min, samples were vortexed and centrifuged at 16,000 g for 5min. Supernatants were transferred to fresh tubes (chromatin-bound fraction).

#### Western blotting

Cells were lysed in RIPA buffer (50mM Tris HCl, pH 7.4, 150mM NaCl, 0.5% deoxycholate, 0.1% sodium dodecyl sulfate, 1% NP-40) containing protease inhibitors (Roche, 11873580001) and 1 μM dithiothreitol (DTT). Whole cell extracts were separated by electrophoresis, transferred onto polyvinylidene difluoride membranes and blocked in 5% skimmed milk dissolved in 0.1%Tween/TBS. Membranes were incubated with primary antibodies (α-BRCA2 (Merck Millipore, ab-1, 1:500), α-BRCA1 (Santa Cruz, C-20, 1:200), α-PALB2 (Bethyl A301-246A, 1:1000), α-RAD51 (GeneTex, 14B4, 1:500), α-p-ATM (S1981) (Abcam, ab81292, 1:1000), α-ATM (Sigma, A1106, 1:500), α-p-ATR (T1989) (GeneTex, GTX128145, 1:500), α-ATR (Bethyl, A300-137A, 1:10000), α-LC3B (Cell Signaling, D11, 1:1000), α-FLAG (Sigma, M2, 1:500), α-polyubiquitin (Enzo, FK2, 1:1000), α-Hsp90 (Cell Signaling, C45G5, 1:1000), α-β-actin (Sigma, A5441, 1:10000), α-XRCC3 (Oncogene, PC691, 1:5000), α-FANCI (Abcam, ab15344, 1:2000), α-Ku80 (Santa Cruz, sc-1485, 1:500), α-Ku70 (Abcam, ab3114, 1:500), α-XRCC4 (Abcam, ab145, 1:2000), α-mCherry (Novus Biologicals, NBP1-96752, 1:2000), α-Histone H3 (Cell Signaling, #9715, 1:1000, α-c-Myc (Santa Cruz, sc-40, 1:500), α-PLK1 (ThermoFisher, 331700, 1:1000), α-Aurora B (Abcam, ab2254, 1:1000), α-TXNL1 (Abcam, ab188328, 1:2000), α-JAK1 (Santa Cruz, sc-376996, 1:500)) overnight at 4°C followed by washing in 0.1%Tween/TBS. Membranes were incubated with appropriate HRP-linked secondary antibodies at 25°C for 1h and washed thrice prior to signal detection. Membranes were developed by chemiluminescence using ECL reagent.

#### Metaphase Spreads

Cells were treated as indicated in the text for 5h, washed three times with media and incubated at 37°C for 18h. Cells were treated with 0.1 μg/ml colcemid (GIBCO 15212-012) for 3-6h and mitotic cells collected. 0.56% KCl solution was slowly added to mitotic cells with gentle mixing and incubated at 37°C for 15min. Three drops of ice-cold Carnoy’s fixative was added to each sample and cells pelleted at 100 g for 5min. Cells were gently resuspended in Carnoy’s fixative and fixed overnight at −20°C. Fixed samples were washed thrice in ice-cold Carnoy’s fixative and spotted onto clean glass slides. Spotted glass slides were held over a beaker of steaming water cells side up for 30 s before air-drying for at least 1h. Chromosomes were stained in Karyomax Giemsa Stain solution (ThermoFisher, 10092013) for 5min, briefly washed twice in Gurr’s buffer, pH 6.8 (ThermoFisher, 10582013) and dried. Slides were mounted in Permount Mounting Medium (VWR, 100496-550). Brightfield images were taken using an Olympus BX51 microscope using 63x or 100x objective lenses.

#### Colony Formation Assay

300,000 cells per well of a 6-well plate were seeded and 24h later, treated with formaldehyde for 5h. Cells were washed with 1X PBS and re-plated at 200 cells/well (HeLa) or 600 cells/well (HBEC) of a 6-well plate in triplicate. 10-14 days later, colonies were washed, fixed with 4% formaldehyde for 20 min at room temperature and stained in 0.1% crystal violet solution (Sigma, HT90132). The number of colonies was manually enumerated.

#### Cycloheximide chase assay

Cells were treated with 100 μg/ml cycloheximide (Sigma, C4859) and in the presence or absence of formaldehyde. DMSO was used as a control for cycloheximide.

#### Measuring Cell Proliferation

5000 cells were seeded in each well of 24-well plates. 24h after seeding, time-lapsed images were obtained using an IncuCyte system (Essen BioScience), with 10x magnification from 9 spots within each well of a 24-well plate every 2 hr over 72 hr. Cell confluency was automatically determined from phase-contrast images at different time points, using the integrated IncuCyte software. The IncuCyte software utilizes a software algorithm that calculates the area occupied by cells as a percentage of the total area of the entire field to give the percentage confluency.

#### Protein extraction and in-solution digestion for SWATH-MS

Cell pellets were suspended in 10M Urea lysis buffer containing complete protease inhibitor cocktail and lysed by sonication at 4°C for 2 min using a VialTweeter device (Hielscher-Ultrasound Technology). Insoluble material was removed by centrifugation at 18,000 g for 1h. Supernatants were reduced by 10mM Tris-(2-carboxyethyl)-phosphine (TCEP) for 1h at 37°C and 20 mM iodoacetamide (IAA) in the dark for 45 min at room temperature. Samples were further diluted by 1:6 (v/v) with 100 mM NH_4_HCO_3_ and digested with sequencing-grade porcine trypsin (Promega) at a protease/protein ratio of 1:25 overnight at 37°C. The amount of purified peptides was determined using Nanodrop ND-1000 (Thermo Scientific) and 1.5 μg peptides were injected in each LC-MS run.

#### SWATH mass spectrometry

Peptide samples after digested were measured by SWATH mass spectrometry with liquid chromatographic (LC) (Collins et al., 2013, Gillet et al., 2012, Liu et al., 2013). Specifically, the mass spectrometer was interfaced with an Eksigent NanoLC Ultra 2D Plus HPLC system. Peptides were directly injected onto a 20-cm PicoFrit emitter (New Objective, self-packed to 20 cm with Magic C18 AQ 3-μm 200-Å material), and then separated using a 90 min gradient from 5%–35% (buffer A 0.1% (v/v) formic acid, 2% (v/v) acetonitrile, buffer B 0.1% (v/v) formic acid, 98% (v/v) acetonitrile) at a flow rate of 300 nL/min. In the present SWATH-MS mode, the SCIEX 5600 plus TripleTOF instrument was specifically tuned to optimize the quadrupole settings for the selection of 64 variable wide precursor ion selection windows. The 64-variable window schema was optimized based on a normal human cell lysate sample, covering the precursor mass range of 400–1,200 m/z. Please refer to Table S3 for the isolation windows. SWATH MS2 spectra were collected from 50 to 2,000 m/z. The collision energy (CE) was optimized for each window according to the calculation for a charge 2+ ion centered upon the window with a spread of 15 eV. An accumulation time (dwell time) of 50 ms was used for all fragment-ion scans in high-sensitivity mode and for each SWATH-MS cycle a survey scan in high-resolution mode was also acquired for 250 ms, resulting in a duty cycle of ∼3.45 s.

### Quantification and Statistical Analysis

In all figures: ns, *p-value* > 0.05; ^∗^, *p-value* < 0.05; ^∗∗^, *p-value* < 0.01; ^∗∗∗^, *p-value* < 0.001. The statistical methods used for comparisons are indicated in the relevant figure legends and in the sections below.

#### DNA fiber analysis

DNA tract lengths were measured using ImageJ. For replication fork stability assays, at least 250 tracts were counted per condition and the Mann-Whitney t test was used to determine *p* values. For sister fork symmetry assays, the ratio of sister forks was achieved by dividing the length of the longer sister CIdU tract by that of the shorter sister CIdU tract which emanate from the same origin of replication. At least 70 sister fork ratios were determined for each sample per experiment. The scatterplots in Figures 1B and 7A show combined results for three and two independent experiments respectively and the Mann-Whitney t test was used to determine statistical significance.

#### Western blot analysis

Densitometric measurements were carried out used Image Studio Lite version 3.1. In Figure 2B, the histogram plots the mean ± SEM from three independent experiments. In Figure 3D, the graph plots the mean ± SEM from three independent experiments and the two-tailed Student’s t test was used to determine statistical significance. In Figures 5A–5C, the histograms plot the mean ± SEM from eight (Figure 5A) and three (Figures 5B and 5C) independent experiments respectively and the two-tailed Student’s t test was used to determine statistical significance in Figure 5A. In Figure S1I, the histogram plots the mean ± SEM from two independent experiments. Where indicated, a single asterisk (^∗^) indicates non-specific bands and a double asterisk (^∗∗^) indicates probable degradation products occurring during sample preparation.

#### Metaphase spread analysis

50 metaphases were analyzed per sample in each experiment. The Mann-Whitney t test was used to determine statistical significance.

#### Colony formation assay analysis

Colonies were manually enumerated and the two-tailed Student’s t test was used to determine statistical significance.

#### Image acquisition and analysis

Stained cells were imaged on a Leica SP5 confocal microscope. Maximum projections of z stacks of each field were generated and analyzed using Cellomics Bioapplication software using Compartment Analysis 4 (Thermo Scientific) algorithm for nuclei segmentation (based on nuclear DAPI stain), PCNA nuclear staining (based on average nuclear intensity), nuclear γH2AX and RAD51 foci counts, EdU and EU staining (based on total nuclear intensity). For Figure 1A, the total nuclear intensity of EdU nuclear staining per cell was determined using nuclear DAPI staining as a mask for nuclear segmentation. The histogram plots the mean ± SEM from three independent experiments. For Figures 1C and 1D, thresholds for nuclei segmentation, PCNA average nuclear intensity, γH2AX foci counts were manually optimized for each independent experiment but kept constant across samples within the same experiment. At least 1500 cells were counted per sample in each experiment. The histograms in Figures 1C and 1D plot the mean ± SEM from four and three independent experiments respectively and the two-tailed Student’s t test was used to determine statistical significance. For Figure S1B, thresholds for nuclei segmentation and RAD51 foci counts were manually optimized for each independent experiment but kept constant across samples within the same experiment. The histogram plots the mean ± SEM from three independent experiments. At least 200 cells were counted per sample in each experiment and the two-tailed Student’s t test was used to determine statistical significance. For Figure S7A, the total nuclear intensity of EU nuclear staining per cell was determined using nuclear DAPI staining as a mask for nuclear segmentation. At least 200 cells per condition were measured.

#### SWATH-MS data analysis

The SWATH-MS identification was performed by OpenSWATH software (Röst et al., 2014) searching against a previously established SWATH assay library which contains mass spectrometric query parameters for 10,000 human proteins with unique Swiss-Prot identities (Rosenberger et al., 2014). OpenSWATH first identified the peak groups from all individual SWATH maps at a target FDR = 1% and then aligned between SWATH maps using a novel TRIC (TRansfer of Identification Confidence) algorithm that was specifically developed for targeted proteomic data analysis (Röst et al., 2016). The re-quantification feature in OpenSWATH was enabled but only those peptide signals detected in at least eight of the ten samples in both FA or control groups were accepted for the protein level quantification, resulting in 34,575 peptide peak groups assigned to 4219 unique SwissProt proteins. The expression data matrix was median normalized (using a simple normalization factor calculated by summing all the peak group signals per sample). To quantify the protein abundance levels across samples, we summed up the most abundant peptides for each protein (i.e., top 3 peptide groups based on intensity were used for those proteins identified with more than three proteotypic peptide signals whereas all the peptides were summarized for other proteins) which allow for reliable estimation of global protein levels (Liu et al., 2015, Ludwig et al., 2012, Wilhelm et al., 2014). The quantitative protein level matrix was then log_2_ transformed for statistical and bioinformatics analysis. The fold changes were calculated based on the normalized SWATH-MS intensities for each protein. The two-tailed Student’s t test was used to determine statistical significance and corrections for multiple hypothesis testing was carried out by Benjamini-Hochberg correction of p values, which are reflected in the volcano plot (Figure 4C).

### Data Availability

Our SWATH-MS proteomics data have been deposited to the ProteomeXchange Consortium via the PRIDE (Vizcaíno et al., 2016) partner repository. The accession number for these data is ProteomeXchange: PXD006106.

## Author Contributions

S.L.W.T. and A.R.V. conceived the work reported in this paper. S.L.W.T. performed the experiments. S. Chadha created the *BRCA2* heterozygous cell lines and assisted in experiments. Y.L. and R.A. performed the SWATH-MS experiments and analyzed them with S.L.W.T., E.G., and A.R.V. D.P., K.A., S. Constantinou, X.R., and M.Y.L. performed experiments to address the reviewers’ comments. S.L.W.T. and A.R.V. analyzed the results and wrote the paper with help from the other authors. A.R.V. supervised this work.

## Supplemental Information

Table S1. Proteins Detected by SWATH-MS with Fold Changes after FA Exposure and the Associated Benjamini-Hochberg Corrected p Values, Related to Figure 4

Table S2. Proteins that Are Reproducibly Depleted by >25% after FA Exposure, Related to Figure 4

Table S3. m/z Ranges of SWATH-MS Isolation Windows, Related to the STAR Methods
